# A 16-Gene Signature Distinguishes Anaplastic Astrocytoma from Glioblastoma

**DOI:** 10.1371/journal.pone.0085200

**Published:** 2014-01-24

**Authors:** Soumya Alige Mahabala Rao, Sujaya Srinivasan, Irene Rosita Pia Patric, Alangar Sathyaranjandas Hegde, Bangalore Ashwathnarayanara Chandramouli, Arivazhagan Arimappamagan, Vani Santosh, Paturu Kondaiah, Manchanahalli R. Sathyanarayana Rao, Kumaravel Somasundaram

**Affiliations:** 1 Department of Microbiology and Cell Biology, Development and Genetics, Indian Institute of Science, Bangalore, India; 2 Department of Molecular Reproduction, Development and Genetics, Indian Institute of Science, Bangalore, India; 3 Sri Sathya Sai Institute of Higher Medical Sciences, Bangalore, India; 4 Department of Neurosurgery, National Institute of Mental Health and Neuro Sciences, Bangalore, India; 5 Department of Neuropathology, National Institute of Mental Health and Neuro Sciences, Bangalore, India; 6 Jawaharlal Nehru Centre for Advanced Scientific Research, Bangalore, India; University of Navarra, Spain

## Abstract

Anaplastic astrocytoma (AA; Grade III) and glioblastoma (GBM; Grade IV) are diffusely infiltrating tumors and are called malignant astrocytomas. The treatment regimen and prognosis are distinctly different between anaplastic astrocytoma and glioblastoma patients. Although histopathology based current grading system is well accepted and largely reproducible, intratumoral histologic variations often lead to difficulties in classification of malignant astrocytoma samples. In order to obtain a more robust molecular classifier, we analysed RT-qPCR expression data of 175 differentially regulated genes across astrocytoma using *P*rediction *A*nalysis of *M*icroarrays (PAM) and found the most discriminatory 16-gene expression signature for the classification of anaplastic astrocytoma and glioblastoma. The 16-gene signature obtained in the training set was validated in the test set with diagnostic accuracy of 89%. Additionally, validation of the 16-gene signature in *multiple* independent cohorts revealed that the signature predicted anaplastic astrocytoma and glioblastoma samples with accuracy rates of 99%, 88%, and 92% in TCGA, GSE1993 and GSE4422 datasets, respectively. The protein-protein interaction network and pathway analysis suggested that the 16-genes of the signature identified epithelial-mesenchymal transition (EMT) pathway as the most differentially regulated pathway in glioblastoma compared to anaplastic astrocytoma. In addition to identifying 16 gene classification signature, we also demonstrated that genes involved in epithelial-mesenchymal transition may play an important role in distinguishing glioblastoma from anaplastic astrocytoma.

## Introduction

Astrocytoma, the tumor of astrocytic glial cells, is one of the common types of primary central nervous system (CNS) neoplasms [1]. It accounts for more than 60% of all primary brain tumors and has a poor prognosis. Diffuse infiltrating astrocytomas are graded into low-grade diffuse astrocytoma (DA; grade II), anaplastic astrocytoma (AA; grade III) and glioblastoma (GBM; grade IV) in the order of increasing malignancy [2]. Patients with diffuse astrocytoma have a median survival time of 6 to 8 years after surgical intervention. The more aggressive anaplastic astrocytoma (AA) and glioblastoma (GBM) are also called malignant astrocytomas [3], [4]. The treatment protocols and length of survival are distinctly different between AA and GBM patients. GBM patients receive radiotherapy and chemotherapy with temozolomide after the surgical removal of the tumor. In case of AA, patients receive radiotherapy after the surgical removal of the tumor tissue. Although AA patients conventionally receive chemotherapy in addition to radiotherapy in some places/countries, chemotherapy failed to consistently improve the outcome of patients with anaplastic astrocytoma [4]. The median survival time for AA patients is 2 to 3 years whereas patients with GBM have a median survival of 12 to 15 months [5], [6] and less than 20% of GBM patients survive two years [7]. GBMs are further divided into primary GBM and secondary GBM on the basis of clinical and molecular profile. Primary GBM presents in an acute *de novo* manner with no evidence of an antecedent lower grade tumor and it accounts for >90% of all GBMs [8]. In contrast, secondary GBM results from the progressive malignant transformation of a DA or AA.

The current WHO grading system of astrocytomas is based on the histopathological characteristics of the underlying tumor tissue. Grading of diffusely infiltrating astrocytomas by individual pathologists is dependent on the specific histologic features: nuclear atypia, mitosis, microvascular proliferation and/or necrosis, which associate with biologically aggressive behaviour (WHO 2007). The prognosis of patients and therapeutic decisions rely on the grading based on pathological features. Though grading based on histology is largely reproducible and well accepted, high-grade astrocytoma tissue samples often have little cellular differentiation, intratumoral histological variability and also can lack identifying histological features [9], [10], [11], [12]. In such cases, precise grading of malignant astrocytoma samples becomes difficult.

Recent studies have attempted to characterize the molecular basis for the histological and prognostic differences between AA and GBM [13], [14], [15], [16], [17]. With the advent of advancements in various expression profiling technologies, efforts are being made to identify molecular markers to refine the current grading method for malignant astrocytoma. Profiling studies have also led to the development of new classification systems based on the molecular profile identified by using various statistical methods [18], [19]. There is a need for a more robust classifier gene set based on molecular profile which would lead to improved classification methods and better management of patients with malignant astrocytoma.

In the present study, we have used gene expression profile in a cohort of 50 AA and 132 GBM patient samples to identify the most discriminatory genes between the two grades. We divided the patient cohort randomly into training and test sets. We then used the prediction analysis of microarrays on the training set to identify a 16-gene signature that could distinguish GBM from AA. Further, the signature was validated in the test set and independent cohorts of AA and GBM patient samples from publicly available datasets. Subsequently, through network analysis using the 16-gene signature, we found that the most differentially regulated pathways between the two grades are related to focal adhesion and the epithelial-mesenchymal transition (EMT), which can possibly explain the more malignant phenotype of grade IV GBMs. Thus, our study demonstrates that gene expression signature, combined with histopathological grading leads to accurate grading of the patient samples, thus helping in prediction of prognosis and deciding most appropriate therapy.

## Materials and Methods

### Patient tumor samples

Tumor samples used in the study were collected from patients who were operated at National Institute of Mental Health and Neurosciences (NIMHANS) and Sri Sathya Sai Institute of Higher Medical Sciences (SSSIHMS), Bangalore, India. For non-neoplastic control brain tissue sample, a portion of the non-dominant anterior temporal cortex resected during surgery for intractable epilepsy was used. Tissues were freshly received from the neurosurgical operating rooms, bisected and one half was placed in RNA later (Ambion Inc., USA), stored at −70°C and was subsequently used for RNA isolation. The other half of the resected tissue was fixed in 10% buffered neutral formalin, processed for paraffin sections and was used for histopathology and immunohistochemistry (IHC). This study has been approved by the ethics committee of NIMHANS (NIMHANS/IEC/No. RPA/060/05 dated 29.10.2005) and SSSIHMS (SSSIHMS/IEC/No RPA/001/2005 dated 20.10.2005) and patient's written consent was obtained. A total of 182 samples of malignant astrocytoma and 20 control brain tissues were used in this study.

### RNA isolation and real-time quantitative reverse transcription-PCR

Total RNA isolation from the frozen tissue and quantification were performed as described before [13]. The relative quantification of the expression levels of selected genes was carried out by real time qPCR. In the first step, cDNA was generated from RNA derived from different tissue samples using a cDNA archive kit (ABI PRISM); subsequently, real-time qPCR was carried out in an ABI PRISM 7900 (Applied Biosystems) sequence detection system with the cDNA as template using gene-specific primer sets and a Dynamo kit containing SYBR green dye (Finnzyme). All measurements were made in triplicates. The genes GARS (glycyl-tRNA synthetase), AGPAT1 (1-acylglycerol-3-phosphate O-acyltransferase 1), ATP5G1 [ATP synthase, H+ transporting, mitochondrial F0 complex, subunit C1 (subunit 9)], and RPL35A (ribosomal protein L35a) were used as internal controls because their expression levels were found to be unaltered in the microarray experiments previously carried out in our laboratory. Non-neoplastic control brain tissue samples from 20 different epilepsy patients were used as reference. Delta Delta Ct method was used for the calculation of ratios of expression levels. Statistical significance was tested by Mann-Whitney test using GraphPad PRISM software. Sequences of reverse transcription-PCR primers are listed in **Table S1 in File S1** and all the primers worked at the annealing temperature of 59°C. For the analysis done as a part of the study, we have used the gene expression data from real time PCR for 50 AAs, 132 GBMs and 20 control non-neoplastic brain tissues. Out of this, real-time qRT-PCR for 50 AAs, 9 GBMs and 20 control non-neoplastic brain samples was carried as a part of the current study while remaining data generation was carried out as a part of a different project.

### Data analysis

Class prediction analysis was performed by Prediction Analysis of Microarrays (PAM) using the PAM package in the R software (version 2.14.2). The method of the nearest shrunken centroids was used to identify a subgroup of genes that best characterizes a predefined class [20]. The prediction accuracy was estimated by 10-fold cross-validation. This means that the data set was divided into 10 approximately equally sized subsets; a PAM model was trained for nine subsets and prediction was conducted for the remaining subset [20]. This training and prediction process was repeated 10 times to include predictions for each subset. Centroid is the average gene expression for each gene in each class divided by the within-class standard deviation for that gene. In the nearest centroid method, a standardized centroid is computed for each class and this is called as class centroid. This method takes the gene expression profile of a new sample and compares it to each of these class centroids. The class whose centroid that it is closest to, in squared distance, is the predicted class for that new sample. Nearest shrunken centroid classification is a modification of the standard nearest centroid classification. In this method, each of the class centroids is “shrunken” towards the overall centroid for all classes by an amount called as the “threshold”. Typically, the user would choose the threshold value giving the minimum cross-validated misclassification error rate.

In Principal Component Analysis (PCA), orthogonal transformation is used to convert a set of variables into a set of values of linearly uncorrelated variables called principal components. The number of principal components is less than or equal to the number of original variables. The first two components account for the largest possible variance in the dataset. PCA was performed using R package (version 2.7.1).

For the TCGA dataset, the level 3 expression data from the Agilent platform was downloaded from the TCGA data portal (*tcga-data.nci.nih.gov/*). For GSE1993, GSE4422 and GSE4271 datasets, the microarray data was downloaded from GEO website (http://www.ncbi.nlm.nih.gov/gds).

### Network analysis

We used the 16 genes in our signature as input genes to the Bisogenet plugin in Cytoscape to generate a protein-protein interaction network. The generated network had 252 nodes (genes) and 1498 edges (interactions between genes/proteins). This network consisted of the seed proteins with their immediate interacting neighbors. We extracted gene expression data for the 252 genes from the GSE1993 dataset. Using the Multi Experiment Viewer (MeV) [21], we performed a t-test with 100 permutations, and chose genes that were differentially regulated FDR corrected p-value<0.1. There were 67 differentially regulated genes, out of which 53 were up regulated and 14 were down regulated. We calculated the fold change for each of these 67 genes and used this data as input for downstream pathway analysis. We used web-based software, the Pathway express, to find pathways that were enriched by the differentially regulated genes. This software looks at the magnitude of the fold change in addition to the genes themselves, to identify the most differentially regulated pathways. From the output of Pathway Express, we chose focal adhesion, which is the most significant pathway to perform further analysis. We extracted gene expression data for all the genes (n = 145) in the KEGG focal adhesion pathway from the GSE1993 dataset. We then identified genes that were differentially regulated between AA and GBM (n = 58) using the MeV software (t-test with 100 permutations FDR corrected p value<0.25).

## Results

### The overall workflow of classification signature identification and validation

**Figure 1:**
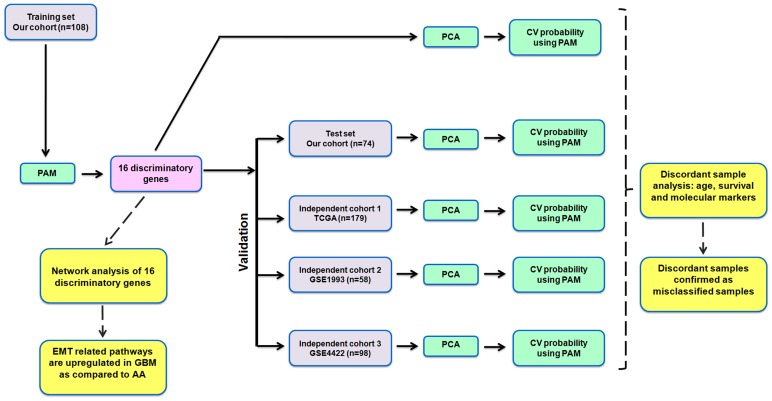
The schematic representation of the work flow of statistical analysis. The expression of 175 genes was subjected to prediction analysis of microarray (PAM) in the training set. PAM analysis identified the 16 discriminatory genes between AA and GBM which was further validated by principal component analysis (PCA) and cross validated probability by PAM. The 16-gene signature was further validated in test set and three independent cohorts of patient samples (GSE1993, GSE4422, TCGA and GSE4271).

In order to identify a gene expression signature to distinguish AA and GBM, we have used the gene expression data of 175 genes. This set of 175 genes were selected based on their differential expression in astrocytoma samples as compared to the control non-neoplastic brain tissue originally derived from previously performed microarray study in our laboratory [16] (**Table S2 in File S1**). In the current study, we have carried out the expression analysis of 175 genes in 50 AA, 9 GBM and 20 non-neoplastic control brain samples by real time RT-qPCR. For GBM cohort, we have used the expression data of 175 genes from an additional set of GBM samples (n = 123) which was carried out as a part of different project. Thus, in total, we have used the expression data from 50 AAs and 132 GBM samples in the present study. First, we randomly divided our patient cohort into training set and test set, each having an equal proportion of AA (27%) and GBM (73%) samples. The schematic of the analysis workflow is shown in **Figure 1**. Next, the expression data from the training set (**Table S2 in File S1**) was analyzed by Prediction Analysis of Microarrays (PAM). In PAM, the method of the nearest shrunken centroids was used to identify a subset of genes that best characterized predefined classes (AA and GBM). PAM analysis identified a 16 gene signature which was further validated through Principal Component Analysis (PCA) and cross validated probability in the test set as well as in independent cohorts of patient samples (TCGA, GSE1993, GSE4422 and GSE4271) from publicly available datasets (**Figure 1**). The number of AA and GBM samples in the training set, test set and independent cohorts is shown in **Table S3 in File S1**.

### Identification of 16-gene classification signature in training set

**Figure 2:**
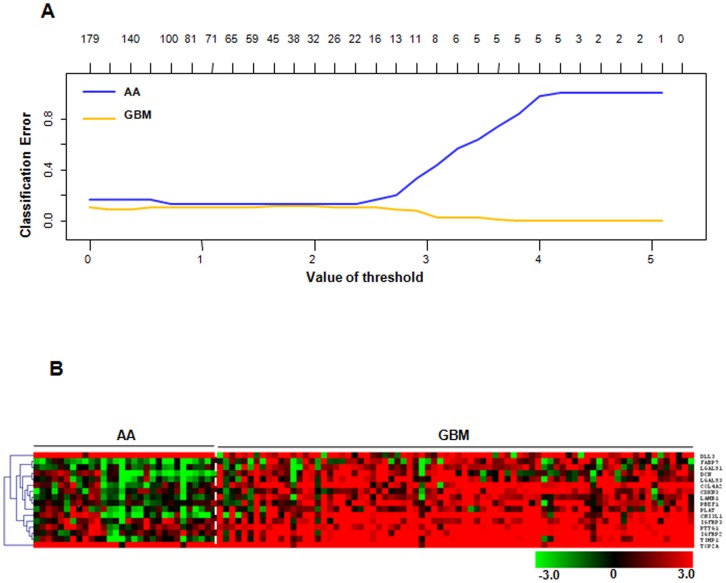
Identification of 16-gene signature in training set. **A.** Plot showing classification error for the 175 input genes from PAM analysis in the training set. The threshold value of 2.5 corresponded to 16 genes which classified AA (n = 30) and GBM (n = 78) samples with classification error of 0.12. **B.** Heat map of one-way hierarchical clustering of 16 PAM-identified genes in AA and GBM patient samples of the training set. A dual-color code was used, with red and green indicating up- and down regulation, respectively.

**Figure 3:**
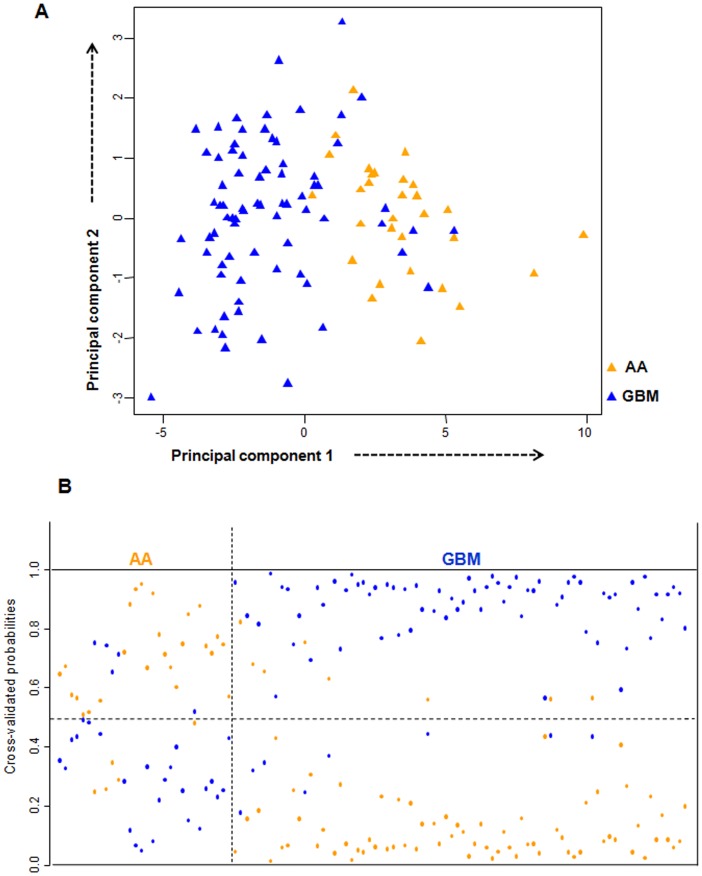
PCA and cross validated probabilities of AA and GBM samples of training set. **A.** PCA was performed using expression values of 16- PAM identified genes between AA (n = 30) and GBM (n = 78) samples in training set. A scatter plot is generated using the first two principal components for each sample. The color code of the samples is as indicated. **B.** The detailed probabilities of 10-fold cross-validation for the samples of training set based on the expression values of 16 genes are shown. For each sample, its probability as AA (orange color) and GBM (blue color) are shown and it was predicted by the PAM program as either AA or GBM based on which grade's probability is higher. The original histological grade of the samples is shown on the top.

Using training set, the PAM analysis identified that, out of 175 genes, 16 genes could discriminate AA and GBM samples at a threshold value 2.5 with an error rate of 0.12 (**Figure 2A**). The 16 genes of the signature were differentially expressed in AA and GBM (**Figure 2B** **and** **Table 1**). We then performed Principal Component Analysis (PCA) of these 16 genes in the training set, and found that the first two principal components were able to distinguish the AA and GBM samples into two distinct groups (**Figure 3A**). Prediction accuracy estimation by 10-fold cross-validation using PAM (**Figure 3B**) revealed that among 30 AA samples, the gene signature predicted 25 samples correctly as AAs (cross-validated probability more than 0.5) with an error rate of 0.16. Similarly among 78 GBM samples analyzed, the signature predicted 70 samples correctly as GBM with an error rate of 0.1(**Figure 3B**). Thus our 16 gene expression signature could discriminate GBMs from AAs with an overall diagnostic accuracy of 87.9% (**Table 2**). The sensitivity of the signature for AA is 80%, whereas for GBM, it is 89.7%; the specificity for AA is 89.7%, whereas for GBM, it is 80% (**Table 2**).

**Table 1:** Expression of 16 genes selected from PAM in AA (n = 30) and GBM (n = 78) samples of the training set.

Gene Symbol	Gene Name	Average of AAs	Average of GBMs	Fold change	P value*
**CDKN3**	Cyclin-dependent kinase inhibitor 3	−0.1	2.0	2.0	3.4×10^−7^
**CHI3L1**	Chitinase 3-like 1	−0.5	4.9	5.5	4.5×10^−13^
**COL4**	Collagen, type IV	−0.5	2.9	3.5	6.9×10^−8^
**DCN**	Decorin	−1.0	1.7	2.7	3.4×10^−6^
**DLL3**	Delta-like 3 protein precursor; delta homolog	7.0	2.7	−4.3	7.1×10^−8^
**FABP7**	Fatty acid binding protein 7	−2.4	0.7	3.1	1.4×10^−7^
**IGFBP2**	Insulin-like growth factor binding protein 2	0.7	4.1	3.4	5.3×10^−14^
**IGFBP3**	Insulin-like growth factor binding protein 3	0.8	3.4	2.6	8.9×10^−8^
**LAMB1**	Laminin, beta 1	−0.5	1.7	2.2	1.1×10^−9^
**LGALS1**	Lectin, galactoside-binding, soluble, 1	−1.2	1.3	2.4	1.8×10^−5^
**LGALS3**	Lectin, galactoside-binding, soluble, 3	−0.8	1.9	2.7	2.4×10^−9^
**PBEF1**	Pre-B-cell colony enhancing factor 1	0.2	2.3	2.1	4.6×10^−11^
**PLAT**	Plasminogen activator, tissue	−0.4	1.8	2.2	1.4×10^−6^
**PTTG1**	Pituitary tumor-transforming 1	1.3	3.4	2.1	5.5×10^−8^
**TIMP1**	Tissue inhibitor of metalloproteinase 1	−0.4	3.6	4.0	1.9×10^−14^
**TOP2A**	Topoisomerase (DNA) II alpha	5.9	8.1	2.2	1.0×10^−5^

* P value from student's T-test.

**Table 2:** The diagnostic accuracy, sensitivity and specificity of 16-gene signature to discriminate AA and GBM in training set, test set and three independent cohorts (TCGA, GSE1993, GSE4422) of patient samples.

Cohort	Dataset	Overall accuracy^A^	Sensitivity^B^	Specificity^C^
**Our cohort**	**Training set**	87.9% (95/108)	AA: 83.3% (25/30), GBM: 89.7% (70/78)	AA: 89.7% (70/78), GBM: 83.3% (25/30)
**Our cohort**	**Test set**	89.1% (66/74)	AA: 90% (18/20), GBM: 88.8% (48/54)	AA: 88.8% (48/54), GBM: 90% (18/20)
**Validation set**	**TCGA**	99.4% (178/179)	AA: 96.3% (26/27), GBM: 100% (152/152)	AA: 100% (152/152), GBM: 96.3% (26/27)
**Validation set**	**GSE1993**	87.9% (51/58)	AA: 73.7% (14/19), GBM: 94.8% (37/39)	AA: 94.8% (37/39), GBM: 73.7% (14/19)
**Validation set**	**GSE4422**	92% (70/76)	AA: 80% (4/5), GBM: 93% (66/71)	AA: 93% (66/71), GBM: 80% (4/5)
**Validation set**	**GSE4271** *	81.6% (80/98)	AA: 54% (10/22), GBM: 89.4% (68/76)	AA: 89.4% (68/76), GBM: 54% (10/12)

A Prediction accuracy was determined by 10-fold cross-validation on malignant astrocytoma samples.

Accuracy = (the number of samples predicted correctly)/(total number of samples analyzed).

B Sensitivity = (the number of positive samples predicted)/(the number of true positives).

C Specificity = (the number of negative samples predicted)/(the number of true negatives).

* 14-genes of the 16-gene signature were used for analysis.

### Validation of 16-gene classification signature in test set

**Figure 4:**
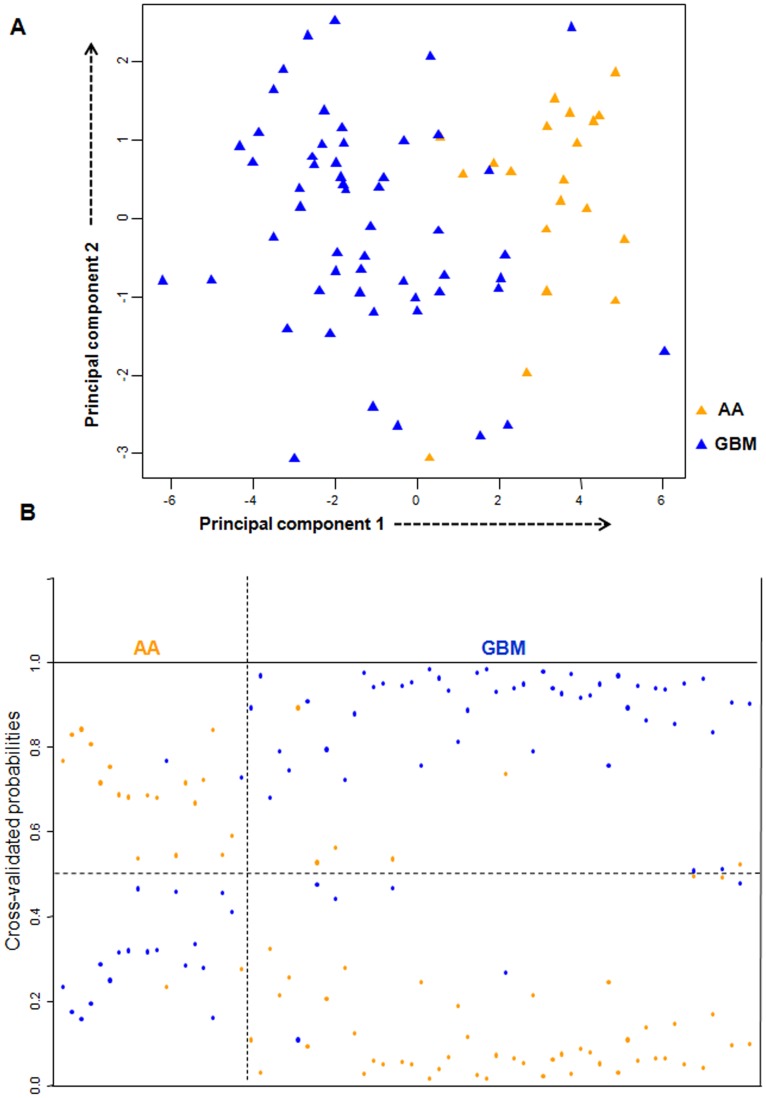
PCA and cross validated probabilities of AA and GBM samples of test set. **A.** PCA was performed using expression values of 16- PAM identified genes between AA (n = 20) and GBM (n = 54) samples in test set. A scatter plot is generated using the first two principal components for each sample. The color of the samples is as indicated. **B.** The detailed probabilities of 10-fold cross-validation for the samples of test set based on the expression values of 16 genes are shown. For each sample, its probability as AA (orange color) and GBM (blue color) are shown and it was predicted by the PAM program as either AA or GBM based on which grade's probability is higher. The original histological grade of the samples is shown on the top.

In the test set, PCA analysis revealed that the expression of 16 genes (as identified from training set) (**Figure S1 and Table S4 in File S1**) was able to distinguish AA from GBM samples (**Figure 4A**). The prediction accuracy estimation by 10-fold cross-validation using PAM (**Figure 4B**) revealed that among 20 AA samples, 16-gene signature predicted 18 samples correctly as AA with an error rate of 0.1. Similarly, among 54 GBM samples, our signature predicted 48 samples correctly as GBM with an error rate of 0.09. Thus the 16 gene expression signature could discriminate GBM from AAs with an overall diagnostic accuracy of 89.1% (**Table 2**). The sensitivity for AA is 90%, whereas for GBM, it is 88.8%; the specificity for AA is 88.8%, whereas for GBM, it is 90% (**Table 2**).

### Additional validation of 16-gene classification signature in independent cohorts

**Figure 5:**
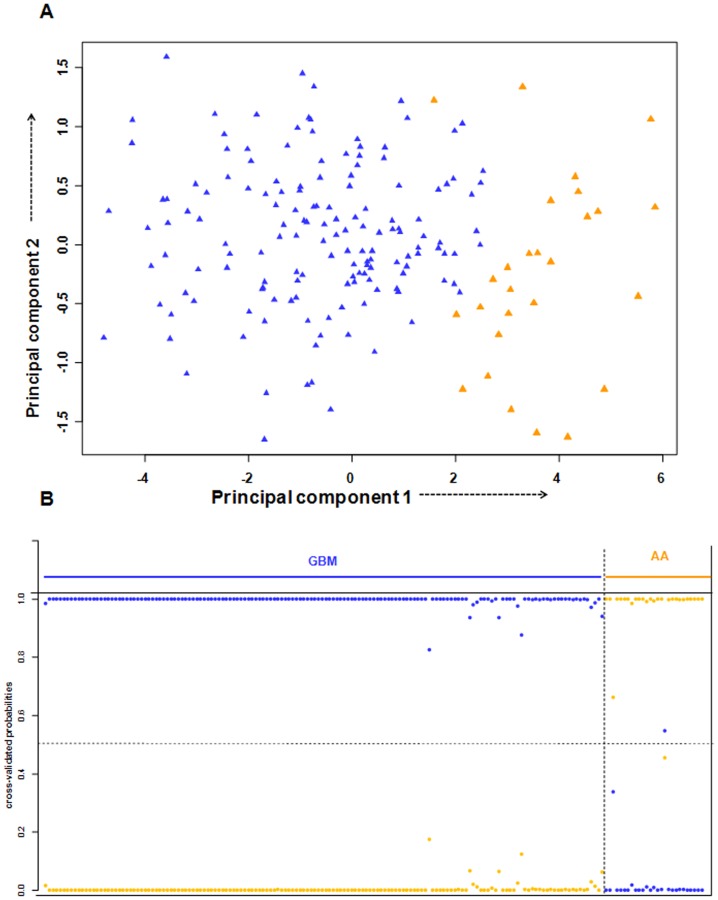
PCA and cross validated probabilities of AA and GBM samples of TCGA dataset. **A.** PCA was performed using expression values of 16- PAM identified genes between grade III glioma (n = 27) and GBM (n = 152) samples in TCGA dataset. A scatter plot is generated using the first two principal components for each sample. The color code of the samples is as indicated. **B.** The detailed probabilities of 10-fold cross-validation for the samples of TCGA dataset based on the expression values of 16 genes are shown. For each sample, its probability as grade III glioma (orange color) and GBM (blue color) are shown and it was predicted by the PAM program as either grade III glioma or GBM based on which grade's probability is higher. The original histological grade of the samples is shown on the top.

To validate the 16-gene signature in independent cohorts of patient samples, we used publicly available microarray datasets: TCGA dataset [22], GSE1993 (from GEO) [19], GSE4422 (from GEO) [23] and GSE4271 (from GEO) [18] datasets. The TCGA dataset [22] comprised of 27 grade III gliomas (comprising of 10 anaplastic astrocytoma, 9 oligoastrocytoma and 8 oligodendroglioma) samples and 152 GBM samples. The 16 genes of the classification signature were differentially expressed in grade III gliomas compared to GBM (**Figure S2 and Table S5 in File S1**). We then performed Principal Component Analysis (PCA) of these 16 genes in the TCGA dataset, and found that the first two principal components were able to distinguish the grade III gliomas and GBM samples into two distinct groups (**Figure 5A**). A10-fold cross validation with PAM using the 16-gene signature was able to classify 26 out of 27 grade III glioma samples and all 152 GBM samples correctly (**Figure 5B**). Thus the 16 gene expression signature could discriminate GBMs from grade III gliomas with an overall diagnostic accuracy of 99.4% (**Table 2**). The sensitivity for grade III glioma is 96.3%, whereas for GBM, it is 100%; the specificity for AA is 100%, whereas for GBM, it is 96.3% (**Table 2**). Additionally, we also used only anaplastic astrocytoma samples (n = 10) along with 152 GBM samples for the PAM analysis and found that the prediction accuracy was 100% (data not shown).

The GSE1993 dataset comprised of 19 AA samples and 39 GBM samples [19]. The PCA analysis of GSE1993 based on the expression of 16 genes (**Figure S3A and Table S6 in File S1**) segregated the samples into distinct AA and GBM subgroups (**Figure S3B in File S1**). Using PAM with a 10-fold cross validation (**Figure S3C in File S1**) the 16-gene signature was able to predict 14 AA samples out of 19 correctly with an error rate of 0.26. Similarly, among 39 GBM samples used, our 16-gene signature predicted 37 samples correctly as GBM with an error rate of 0.05 (**Figure S3C in File S1**). Thus, the 16 gene expression signature could discriminate GBM from AAs with an overall diagnostic accuracy of 87.9% (**Table 2**). The sensitivity for AA is 73.9%, whereas for GBM, it is 94.8%; the specificity for AA is 94.8%, whereas for GBM, it is 73.9% (**Table 2**).

Another independent dataset GSE4422 contained 5 AA and 71 GBM samples [23]. PCA based on the expression of our 16 gene signature (**Figure S4A and Table S7 in File S1**), segregated the samples into distinct AA and GBM subgroups (**Figure S4B in File S1**). Using PAM with 10-fold cross-validation, (**Figure S4C in File S1**) our 16 gene signature was able to classify 4 out of 5 AA samples with error rate of 0.2 and 66 out of 71 GBM samples correctly with an error rate of 0.07 (**Figure S4C in File S1**). Thus the 16-gene expression signature could discriminate GBMs from AAs with an overall diagnostic accuracy of 92% (**Table 2**). The sensitivity for AA is 80%, whereas for GBM, it is 93%; the specificity for AA is 93%, whereas for GBM, it is 80% (**Table 2**).

The validation of the 16-gene signature in GSE4271 dataset (Phillips dataset) revealed that the overall accuracy of the 16-gene signature in this dataset was 82%, however the sensitivity for AA samples was low (54%). Though the 16-gene signature was validated with high accuracy rates in three independent cohorts, in GSE4271 dataset, the accuracy was lower. The reason for the lower accuracy of the signature for AA samples in this dataset is described later in the discussion section. The details of the analysis are provided in the **Supplementary Results in File S1** section along with **Figure S5 in File S1**.

### Comparison of previously reported signatures with the 16-gene classification signature

As the previous report by Petalidis *et al* identified a 59-gene signature for the subtyping of high grade astrocytoma, which correlated with survival of the patients, we checked the 59-genes in classifying AA and GBM samples. For this analysis, we have chosen the TCGA dataset. The detailed analysis is described in the **Supplementary Results in File S1** section along with **Figure S6 in File S1**. The analysis with 59-gene signature showed that the set of 59-genes was able to classify AA and GBM samples of the TCGA dataset accurately, with 100% accuracy. This showed that the 59-gene signature of the Petalidis *et al* and the 16-gene signature both worked equally well for the classification of AA and GBM.

We performed additional analysis also to assess the potentiality of the Phillips *et al* gene signature [18] in classification of high gliomas. The Phillips *et al* gene signature is meant for the prediction of the survival in high-grade glioma [18]. The potential use of Phillips *et al* gene signature for high grade classification was tested mainly because of the reason that grade III gliomas were represented in Proneural subtype that had the best survival among the three subtypes. Our analyses of Phillips *et al* gene-signature across various datasets suggest that the Phillips gene signature fails to consistently predict AA and GBM samples with high accuracy. The sensitivity of the signature for AA varies greatly across datasets: 0% in GSE4422 dataset **Figure S9 in File S1**; 44% in our dataset **Figure S7 in File S1**, 50% in GSE4271 dataset (Phillips dataset) **Figure S8 in File S1** and 85% in TCGA dataset **Figure S10 in File S1**. In particular, the Phillips gene signature fails to predict AA samples accurately, thus compromising the sensitivity for AA prediction and specificity for GBM prediction. A possible reason for the inability of Phillips gene signature to classify AAs from GBMs is that the signature was not developed for this purpose. The detailed account of the analysis is given in the **Supplementary Results in File S1** section along with **Figure S7, Figure S8, Figure S9 and Figure S10 in File S1**.

### The discordant AA and GBM samples identified by the 16-gene signature had GBM-like or AA-like features respectively

Since the 16-gene signature identified the discordant samples, whose grading did not match with the original histopathological grading, it was of our interest to find out whether discordant samples have clinical features and genetic markers similar to that of grades identified by 16-gene signature. For this purpose, we analyzed clinical features like age and survival as well as the molecular markers like CDKN2A/2B loss, EGFR amplification and p53 mutation. For these analyses, we divided the patients into four groups: Authentic AA (samples which were identified as AA by histopathology as well as by 16-gene signature), Authentic GBMs (samples which were identified as GBM by histopathology as well as by 16-gene signature), Discordant AA (samples identified as AA by histopathology but having a GBM-like profile as per the 16-gene signature) and Discordant GBMs (samples identified as GBM by histopathology but having an AA-like profile as per the 16-gene signature).

**Figure 6:**
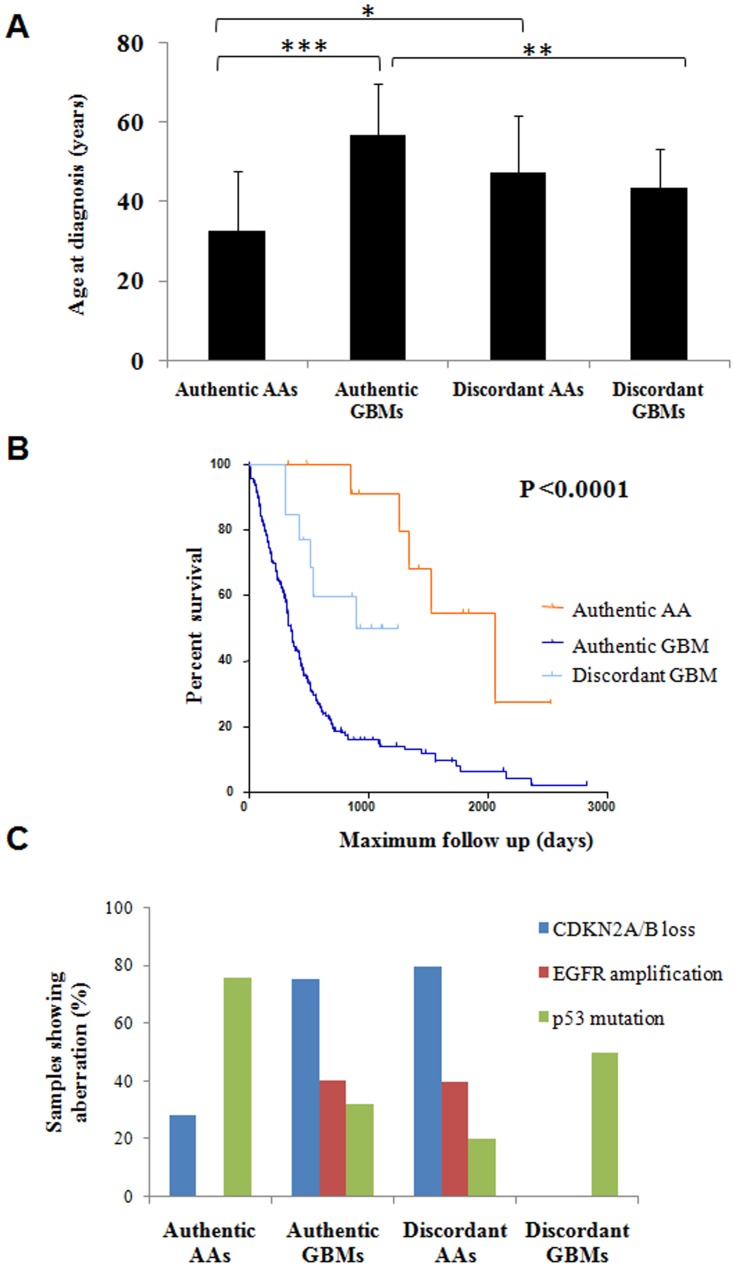
Discordant samples do not exhibit clinical features and molecular markers of its histologic grade. **A.** The average Age at Diagnosis along with standard deviation is plotted for Authentic AAs (n = 21), Authentic GBMs (n = 37), Discordant AAs (n = 8) and Discordant GBMs (n = 20). *** denotes that P<0.001, ** denotes that P<0.01 and * denotes that P<0.05. **B.** The Kaplan Meier survival analysis of Authentic AAs (n = 13), Authentic GBMs (n = 165) and Discordant GBMs (n = 13). The median survival was significantly different across the groups with P<0.001. **C.** The percentage of samples showing CDKN2A/2B loss, EGFR amplification and p53 mutation is plotted for Authentic AAs (n = 14), Authentic GBMs (n = 37), Discordant AAs (n = 5) and Discordant GBMs (n = 2).

With respect to the age at diagnosis, as expected [5], the average age of Authentic AAs (n = 21; average age being 33 years) and Authentic GBMs (n = 37; average age being 56.8 years) were significantly different (P = 0.3×10^−6^) (**Figure 6A**). In case of Discordant AAs (n = 8), the average age was 47.5 years, which was found to be significantly higher than that of Authentic AAs (P = 0.03) while it was similar to that of Authentic GBMs (P = 0.12) (**Figure 6A**). Similarly, in case of Discordant GBMs (n = 13), the average age was 43.6 years which was found to be significantly less than that of Authentic GBMs (P = 0.0006). However, the difference between the Discordant GBMs and Authentic AAs were also found to be significantly different (P = 0.01) (**Figure 6A**).

We further compared the survival of patients across the four groups. The Authentic AA samples (n = 13; median survival of 5.7 years) had significantly better survival when compared with the Authentic GBMs (n = 165; median survival of 0.96 years) with P value of <0.0001 as expected [5] (**Figure 6B**). The Discordant GBMs (n = 13; median survival of 2.46 years) had better survival as compared to Authentic GBMs (n = 165; median survival of 0.96 years) with P value of 0.008 (**Figure 6B**). However, the difference in survival between the Discordant GBMs and Authentic AAs were also found to be significantly different (P = 0.01) (**Figure 6B**). Since there was only one Discordant AA, it was not included in the analysis. However, it is important to note that this patient survived only for 1.2 years.

We further looked at the molecular markers like CDKN2A/2B loss and EGFR amplification, which are characteristic of GBMs [49] and p53 mutation which is characteristic of AAs [49] across the four groups of samples of GSE1993 dataset [19] (**Figure 6C**). CDKN2A/2B loss was found in 75% of Authentic GBMs (n = 37) and 28% of Authentic AAs (n = 21). In case of Discordant AAs (n = 5), 80% of the samples showed CDKN2A/2B loss suggesting the presence of characteristic GBM-like feature in Discordant AA samples (**Figure 6C**). Similarly, when we looked at EGFR amplification, another characteristic feature of GBM, 40% of the Authentic GBMs (n = 37) showed the amplification of EGFR gene whereas none of the Authentic AAs showed EGFR amplification. In case of Discordant AAs (n = 5), 40% of the samples showed EGFR amplification which again suggested that Discordant AAs indeed had GBM-like features (**Figure 6C**). p53 mutation, which is characteristic of AA, was present in 76% of Authentic AAs and 32% of Authentic GBMs. Only 20% of the Discordant AA samples showed p53 mutation whereas 50% of the Discordant GBMs showed p53 mutation which suggested that Discordant AAs less frequently had p53 mutation whereas more Discordant GBMs had p53 mutation (**Figure 6C**). These results together revealed that discordant AA and discordant GBM samples had both clinical features and genetic markers similar to that of GBMs and AAs respectively. This suggests that the 16-gene signature could identify a subset within histologically diagnosed AA having clinical and genetic features of GBM and vice versa, perhaps due to genetic heterogeneity.

### Network analysis identifies differentially regulated pathways

**Figure 7:**
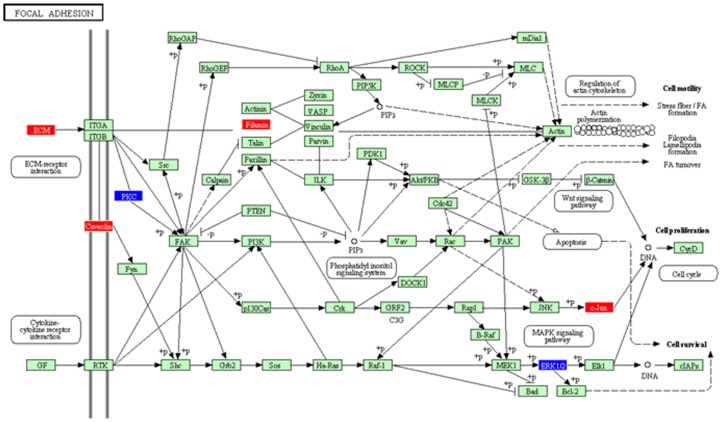
KEGG pathway analysis showed the upregulation of Focal adhesion pathway in GBM. 67 differentially expressed genes between AA and GBM from GSE1993 was subjected to network analysis in Pathway Express which identified the focal adhesion as the significantly differentially regulated pathway between AA and GBM. The input genes present in the network are represented in red and blue indicating the upregulation or downregulation respectively in GBM as compared to AA.

In order to identify the pathways that 16 genes of the classification signature could regulate or associated with, we used the 16 genes as seed proteins to construct a protein-protein interaction network using the Bisogenet plugin in Cytoscape. The resulting network had 252 nodes representing genes and 1498 edges, representing interactions (**Figure S11 in File S1**). Out of these 252 genes, we identified a subset of 67 genes that were differentially regulated genes between GBM and AA (p<0.05 and FDR<0.1). We used these genes along with their fold changes as input into the Pathway Express tool from *K*yto *E*ncyclopedia of *G*enes and *G*enomes (KEGG) to identify differentially regulated pathways between AA and GBM. KEGG pathway identified 10 pathways as differentially regulated between GBM and AA with significance (**Table 3**). Among these pathways, the most significant ones with the maximum number of input genes playing a role in them, were ECM (10 input genes) and focal adhesion (14 input genes) (**Figure 7** **. and** **Table 3** **.**). These pathways are known to be involved in the epithelial to mesenchymal transition (EMT) which is a hallmark of more aggressive tumors like GBM [5]. This further emphasized the fact that the genes in our signature along with their interacting partners were involved in the epithelial to mesenchymal transition in GBM tumors, which explains the aggressiveness and highly infiltrative nature of GBM tumors in comparison to AA tumors.

**Table 3:** Top differentially regulated KEGG pathways between AA and GBM as identified by Pathway Express.

Rank	Pathway Name	Impact Factor	No. of genes in pathway	No. of input genes in pathway	corrected gamma p-value
1	ECM-receptor interaction	32.3	84	10	3.12E-13
2	Focal adhesion	31.56	203	14	6.41E-13
3	Pathways in cancer	25.38	330	12	2.52E-10
4	Small cell lung cancer	18.48	86	6	1.84E-07
5	MAPK signaling pathway	11.68	272	6	1.08E-04
6	Bladder cancer	10.96	42	3	2.08E-04
7	Cell cycle	10.88	118	4	2.25E-04
8	Complement and coagulation cascades	10.49	69	3	3.20E-04
9	GnRH signaling pathway	10.42	103	4	3.40E-04
10	TGF-beta signaling pathway	9.4	87	3	8.60E-04

## Discussion

The standard therapeutic protocol for GBM patients includes maximal safe resection of the tumor mass followed by radiotherapy with concomitant, and further, cyclical chemotherapy (Temozolomide) [3]. Some studies have shown a small, but significant improvement in survival of AA patients with chemotherapy in addition to irradiation [24], [25]. However, improvement in the outcome of AA patients with chemotherapy was not consistent [4]. The length of survival is distinctly different between AA and GBM patients [6]. Because of the intratumoral histologic variations, there is a need for molecular classification based on gene expression.

In the present study, using the expression data of 175 genes-which were found to be associated with glioma before in cohort of AA and GBM patient samples, we identified a 16-gene classification signature in the training set having 30 AA and 78 GBM samples. In the training set, the 16-gene classification signature predicted AA and GBM samples with accuracy of 87.9%. The signature was further validated in test set comprising of 20 AAs and 54 GBMs. In the test set, the expression of 16 genes predicted the grading with accuracy of 89.1%. Interestingly, one of the GBM outliers of the test set, GS 246, was identified as discordant as per the microRNA expression signature identified previously [17]. This indicates that the molecular profiles are better able to classify the samples and are also consistent.

We also validated the expression signature in four publicly available datasets: TCGA [22], GSE1993 [19] GSE4422 [23] and GSE4271 [18]. In TCGA dataset, the 16-gene signature was validated in sample set of 27 grade III gliomas and 152 GBM samples. In this dataset, the prediction accuracy was 99.4%. The specificity for AA and sensitivity for GBM was 100% suggesting the robustness of the classification signature. In GSE1993 dataset, there were 19 AA and 39 GBM samples. The 16-gene signature predicted the classification with an accuracy of 87.9%. The additional validation of the 16-gene signature in GSE4422 identified that the signature predicted the AA and GBM samples with accuracy of 92.7%. However, the overall accuracy of the signature in GSE4271 was 82%, while the sensitivity of the signature for AA samples was 54.5%. While we do not know the exact reason for the low sensitivity of 16 gene signature for AA in GSE4271 dataset, one possible reason could be because of the missing data for 2 genes (DCN and LGALS3; see **Supplementary Results in File S1** for details) of the 16-gene signature.

In three independent datasets (TCGA, GSE1993 and GSE4422), the 16-gene signature was validated with accuracy rate of 88% to 99%. However, there was a small proportion of the discordant samples in each of the datasets. Further to verify, if the clinical features and the molecular markers (specific for AA and GBM) correlate with the grading done by 16-gene signature, we have performed additional analysis on age, survival and molecular markers (CDKN2A/2B loss, EGFR amplification and p53 mutation). This analysis clearly demonstrated that AA discordant samples had GBM-like features and GBM discordant samples had AA-like features.

The aggressive nature of the glioblastoma is due to the highly invasive tumor cell population that invades to normal brain tissue. High infiltrative nature of the glioblastoma is considered to be the important factor contributing to their dismal outcomes. EMT is considered as major modulator of metastasis in epithelial solid tumors; whereas in case of tumors of neuroepithelial type observations pointing towards an EMT(-like) process have been recently reported [47]. EMT-like changes in malignant gliomas are attributed to TWIST1, ZEB1/ZEB2 and SNAIl1/SNAIl2 as inducers for cell-invasiveness in GBMs [48].

With the intention of finding out the differentially regulated pathways by the 16 genes of the 16-gene signature, we used the 16 genes as seed proteins to generate a protein-protein interaction network. We then identified only those genes in the network that were differentially regulated between GBM and AA and used them for downstream pathway analysis. Interestingly, we found that the most differentially regulated pathways were related to the epithelial to mesenchymal transition (EMT), which is known to contribute to the aggressiveness of GBMs compared to lower grades of glioma. Since 16 genes of our classification signature directly or through interactions play a role in EMT pathway, they were able to accurately distinguish GBM tumors from less malignant AA tumors. This also explains why our signature was extremely successful in the precise classification of Grade IV tumors from Grade III tumors.

Several groups have previously reported the global transcript profiling of glioma patient samples comprising of different grades of astrocytoma and oligodendroglioma. While majority of the reports highlighted the identification of grade specific gene expression, few of them aimed at classifying the samples based on the differential gene expression across different grades. Some of these studies identified the gene expression signature which better correlated with the prognosis of the patients than the histopathological grading.

Various studies based on the microarray expression profiling have identified the differentially regulated genes across different grades of glioma [13], [14], [16], [26], [27], [28]. Gene expression profiling by Liang *et al* revealed large expression differences between GBMs and lower-grade oligodendroglial tumors [12]. Importantly, the gene expression patterns in paired specimens of same GBM that were having strikingly divergent histologies were more closely related to each other than to any other tumor [12]. Large scale gene expression profiling of GBM and comparison with lower-grade astrocytomas led to the identification of glioblastoma-associated genes (GAG) that were associated with more malignant phenotype of GBM [15]. Further analysis defined expression differences between pGBM and sGBM. Secondary GAGs suggested the loss of function in prominent cell cycle regulators, whereas primary GAGs highlighted that played important role in extracellular signaling. To compile the expression profiling done by various groups, meta-analysis was done using publicly available genome-scale mRNA datasets comprising of AA and GBM samples [29]. Using the rank sum approach, >20 significant Biocarta pathways were selected, of which the hypoxia-inducible factor (HIF) pathway had highest significance suggesting that many of the statistically significant genes work together in a HIF1A/VEGF-regulated network to increase angiogenesis and invasion in GBM as compared to AA.

Using the expression profiling data, Phillips *et al* identified proneural, mesenchymal and proliferative as three prognostic subclasses of high grade astrocytoma [18]. Using artificial neural network (ANN) for microarray gene expression data for AA and GBM samples, 59-classifier genes were identified and these genes characterized three molecular tumor subtypes [19]. Three identified tumor subtypes correlated better with prognosis than histopathological grading, indicating a high prognostic potential for the 59 gene classifiers. Gene expression studies also revealed that the classifications based on the molecular profiles are useful not only in accurate diagnosis but also in predicting the clinical outcome than standard pathology [23], [30].

Several genes of the 16-gene signature were found to be individually associated with glioma. The aberrant splicing of CDKN3 (cyclin-dependent kinase inhibitor 3) mRNA in human gliomas generated a dominant negative CDKN3 variant and the CDKN3 over expression correlated with decreased patient survival [31]. CHI3L1 (YKL-40) expression levels in tissue and serum correlated with poor prognosis of GBM patients [32], [33]. Subsequently it was shown to upregulate VEGF and to promote angiogenesis and radioresistance, thus promoting the progression of glioblastoma [34]. COL4A2 was found to be over expressed in AA and GBM as compared to DA and was also amplified in GBM samples [26], [35]. DCN (decorin) was known as tumor suppressor in glioma [36], [37], [38] but it was found to be up regulated in our patient cohort as well as in GSE1993, GSE4422 and TCGA datasets. Higher expression of FABP7 was shown to negatively correlate with survival of GBM patients [12] and the over expression of FABP7 increased the migration of glioma cells *in vitro*. Over expression of Insulin-like growth factor binding proteins-2,3 was reported in glioma [39], [40], [41] and it is known to be a modulator of the action of insulin-like growth factors (IGFs), whereas IGF-independent effects of IGFBP-2 on cellular proliferation, apoptosis, and mobility have also been revealed [41]. Laminin was found to be up regulated and correlated with poor prognosis of GBM patients [42]. Inhibition of laminin 8 in glioma cells reduced the invasiveness of glioma cells [42]. NAMPT (PBEF1) is shown to be up regulated in malignant astrocytoma and also in serum samples of the patients [43], [44]. PBEF1 expression in the tumor tissue along with its co-expression with p53 was associated with poor survival [16]. PTTG (Pituitary tumor-transforming gene) which is known to function in the control of mitosis and DNA repair and markedly increased expression of PTTG was found in high-grade gliomas compared to low-grade gliomas and associated with an unfavorable patient outcome [45]. TOP2A (Topoisomerase 2A) was found to be upregulated in glioblastoma compared to the lower grades and the mRNA levels of TOP2A correlated with the better survival in glioblastoma patients [46].

Our finding that the 16-gene expression signature could accurately discriminate GBM from AA tumors raises the possibility of using the expression signature to develop rapid and accurate molecular diagnostic test to distinguish AA and GBM in the future. The additional validation of the signature in the independent cohorts of patient samples suggests the consistency of the 16-gene signature in accurate prediction of AA and GBM samples. The 16 gene signature can help in identifying the subsets in histologically diagnosed AA and GBM, which would actually behave different. Furthermore, when there is an ambiguity in histological diagnosis, the 16 gene signature can help to classify the tumor and predict the prognosis. The functional characterization of the 16-genes of the classification signature in glioma cell lines and mouse models might throw light on their biological importance in astrocytoma development and progression.

## Supporting Information

File S1 **Supplementary Text: Supplementary Methods and Supplementary Results. Supplementary Tables: Table S1.** Primers used for RT-qPCR. **Table S2.** List of genes selected for expression analysis by PCR array. **Table S3.** Number of AA and GBM patient samples in training set, test set and three independent cohorts of patient samples (TCGA, GSE1993 and GSE4422). **Table S4.** Expression of 16 genes in AA (n = 20) and GBM (n = 54) samples of the test set. **Table S5.** Expression of 16 genes in Grade III glioma (n = 27) and GBM (n = 152) samples of the TCGA dataset. **Table S6.** Expression of 16 genes in AA (n = 19) and GBM (n = 39) samples of GSE1993 dataset. **Table S7.** Expression of 16 genes in AA (n = 5) and GBM (n = 71) samples of the GSE4422 dataset. **Supplementary Figures: Figure S1.** Heat map of one-way hierarchical clustering of 16 PAM-identified genes in AA (n = 20) and GBM (n = 54) patient samples in the test set. A dual-color code was used, with red and green indicating up- and down regulation, respectively. **Figure S2.** Heat map of one-way hierarchical clustering of 16 PAM-identified genes in grade III glioma (n = 27) and GBM (n = 152) patient samples in TCGA dataset. A dual-color code was used, with red and green indicating up- and down regulation, respectively. **Figure S3.** **A.** Heat map of one-way hierarchical clustering of 16 PAM-identified genes in AA (n = 19) and GBM (n = 39) patient samples in GSE1993 dataset. A dual-color code was used, with red and green indicating up- and down regulation, respectively. **B.** PCA was performed using expression values of 16-PAM identified genes between AA and GBM samples in GSE1993 dataset. A scatter plot is generated using the first two principal components for each sample. The color of the samples is as indicated. **C.** The detailed probabilities of 10-fold cross-validation for the samples of GSE1993 dataset based on the expression values of 16 genes are shown. For each sample, its probability as AA (orange color) and GBM (blue color) are shown and it was predicted by the PAM program as either AA or GBM based on which grade's probability is higher. The original histological grade of the samples is shown on the top. **Figure S4.** **A.** Heat map of one-way hierarchical clustering of 16 PAM-identified genes in AA (n = 5) and GBM (n = 71) patient samples in GSE4422 dataset. A dual-color code was used, with red and green indicating up- and down regulation, respectively. **B.** PCA was performed using expression values of 16-PAM identified genes between AA and GBM samples in GSE4422 dataset. A scatter plot is generated using the first two principal components for each sample. The color of the samples is as indicated. **C.** The detailed probabilities of 10-fold cross-validation for the samples of GSE4422 dataset based on the expression values of 16 genes are shown. For each sample, its probability as AA (orange color) and GBM (blue color) are shown and it was predicted by the PAM program as either AA or GBM based on which grade's probability is higher. The original histological grade of the samples is shown on the top. **Figure S5.** A. The detailed probabilities of 10-fold cross-validation for the samples of GSE4271 dataset based on the expression values of 16 genes are shown. For each sample, its probability as AA (orange color) and GBM (blue color) are shown and it was predicted by the PAM program as either AA or GBM based on which grade's probability is higher. The original histological grade of the samples is shown on the top. **B.** The average Age at Diagnosis along with standard deviation is plotted for Authentic AAs (n = 12), Authentic GBMs (n = 68), Discordant AAs (n = 10) and Discordant GBMs (n = 8) of GSE4271 dataset. **C**. The Kaplan Meier survival analysis of samples of GSE4271 dataset. **Figure S6.** PAM analysis of the Petalidis-gene signature in TCGA dataset. **A.** Plot showing classification error for the Petalidis gene set in TCGA dataset. The threshold value of 0.0 corresponded to all 54 genes which classified AA (n = 27) and GBM (n = 604) samples with classification error of 0.000. **B**. The detailed probabilities of 10-fold cross-validation for the samples of TCGA dataset based on Petalidis gene set are shown. For each sample, its probability as AA (green color) and GBM (red color) are shown and it was predicted by the PAM program as either AA or GBM based on which grade's probability is higher. The original histological grade of the samples is shown on the top. **Figure S7.** PAM analysis of the Phillips gene signature in our dataset. **A.** Plot showing classification error for the Phillips gene set in our dataset. The threshold value of 0.0 that correspond to all 5 genes which classified AA (n = 50) and GBM (n = 132) samples with classification error of 0.159. **B.** The detailed probabilities of 10-fold cross-validation for the samples of our dataset based on Phillips gene set are shown. For each sample, its probability as AA (orange color) and GBM (blue color) are shown and it was predicted by the PAM program as either AA or GBM based on which grade's probability is higher. The original histological grade of the samples is shown on the top. **Figure S8.** PAM analysis of the Phillips gene signature in Phillips dataset. **A.** Plot showing classification error for the Phillips gene set in Phillips dataset. The threshold value of 0.0 that correspond to all 8 genes which classified AA (n = 24) and GBM (n = 76) samples with classification error of 0.169. **B.** The detailed probabilities of 10-fold cross-validation for the samples of our dataset based on Phillips gene set are shown. For each sample, its probability as AA (orange color) and GBM (blue color) are shown and it was predicted by the PAM program as either AA or GBM based on which grade's probability is higher. The original histological grade of the samples is shown on the top. **Figure S9.** PAM analysis of the Phillips gene signature in GSE4422 dataset. **A.** Plot showing classification error for the Phillips gene set in GSE4422 dataset. The threshold value of 0.0 that correspond to all 8 genes which classified AA (n = 5) and GBM (n = 76) samples with classification error of 0.065. **B.** The detailed probabilities of 10-fold cross-validation for the samples of our dataset based on Phillips gene set are shown. For each sample, its probability as AA (orange color) and GBM (blue color) are shown and it was predicted by the PAM program as either AA or GBM based on which grade's probability is higher. The original histological grade of the samples is shown on the top. **Figure S10.** PAM analysis of the Phillips-gene signature in TCGA dataset. **A.** Plot showing classification error for the Phillips gene set in TCGA dataset. The threshold value of 0.0 corresponded to all 8 genes which classified AA (n = 27) and GBM (n = 604) samples with classification error of 0.008. **B.** The detailed probabilities of 10-fold cross-validation for the samples of TCGA dataset based on Phillips gene set are shown. For each sample, its probability as AA (orange color) and GBM (blue color) are shown and it was predicted by the PAM program as either AA or GBM based on which grade's probability is higher. The original histological grade of the samples is shown on the top. **Figure S11.** Network obtained by using 16-genes of classification signature as input genes to Bisogenet plugin in Cytoscape. The gene rated network had 252 nodes (genes) and 1498 edges (interactions between genes/proteins). This network consisted of the seed proteins with their immediate interacting neighbors. The nodes corresponding to the input genes are highlighted by the bigger node size as compared to the rest of the interacting partners. The color code is as indicated in the scale.
(PDF)
