# Supplementary material for: A 16-Gene Signature Distinguishes Anaplastic Astrocytoma from Glioblastoma
Source: PLoS One. 2014 Jan 24;9(1):e85200. doi: 10.1371/journal.pone.0085200 (PMC3901657; doi:10.1371/journal.pone.0085200)
Supplement: File S1 — Supplementary Text: Supplementary Methods and Supplementary Results. Supplementary Tables: Table S1. Primers used for RT-qPCR. Table S2. List of genes selected for expression analysis by PCR array. Table S3. Number of AA and GBM patient samples in training set, test set and three independent cohorts of patient samples (TCGA, GSE1993 and GSE4422). Table S4. Expression of 16 genes in AA (n = 20) and GBM (n = 54) samples of the test set. Table S5. Expression of 16 genes in Grade III glioma (n = 27) and GBM (n = 152) samples of the TCGA dataset. Table S6. Expression of 16 genes in AA (n = 19) and GBM (n = 39) samples of GSE1993 dataset. Table S7. Expression of 16 genes in AA (n = 5) and GBM (n = 71) samples of the GSE4422 dataset. Supplementary Figures: Figure S1. Heat map of one-way hierarchical clustering of 16 PAM-identified genes in AA (n = 20) and GBM (n = 54) patient samples in the test set. A dual-color code was used, with red and green indicating up- and down regulation, respectively. Figure S2. Heat map of one-way hierarchical clustering of 16 PAM-identified genes in grade III glioma (n = 27) and GBM (n = 152) patient samples in TCGA dataset. A dual-color code was used, with red and green indicating up- and down regulation, respectively. Figure S3. A. Heat map of one-way hierarchical clustering of 16 PAM-identified genes in AA (n = 19) and GBM (n = 39) patient samples in GSE1993 dataset. A dual-color code was used, with red and green indicating up- and down regulation, respectively. B. PCA was performed using expression values of 16-PAM identified genes between AA and GBM samples in GSE1993 dataset. A scatter plot is generated using the first two principal components for each sample. The color of the samples is as indicated. C. The detailed probabilities of 10-fold cross-validation for the samples of GSE1993 dataset based on the expression values of 16 genes are shown. For each sample, its probability as AA (orange color) and GBM (blue color) are shown and it w [file pone.0085200.s001.pdf]

Supporting Information

Index

Supplementary Text: page numbers 1-6

Supplementary Tables: page numbers 7-21

Supplementary Figures: page numbers 21-39

Supplementary Methods

Selection of patient cohort for the analysis of age, survival and molecular markers of discordant samples.

To additionally analyze the clinical features like age and survival as well as the molecular markers, we combined the patients of our cohort (patients of training and test set) as well as those of validation datasets (TCGA, GSE1993 and GSE4422) based on the on the availability of the information. For Age, we the included Authentic AA samples (n=21) and Authentic GBM (n=37) samples of GSE1993 dataset. A total of 8 Discordant AA samples were from our cohort (n=2), GSE1993 dataset (n=5) and GSE4422 dataset (n=1). A total of 20 Discordant GBM samples were from our cohort (n=13), GSE1993 dataset (n=2) and GSE4422 dataset (n=5). For survival analysis, samples of GSE1993 dataset were not included because of lack of censoring status. A total of 13 Authentic AAs were from TCGA (n=9) and GSE4422 (n=4) datasets. For Authentic GBMs, a total of 165 samples were considered from GSE4422 (n=66) and TCGA datasets (n=99). For Discordant GBM samples, a total of 13 samples were considered from our patient cohort (n=8) and GSE4422 dataset (n=5). Since information on CDKN2A/2B loss, EGFR amplification and p53 mutation was available for samples of GSE1993 dataset, Authentic and Discordant samples of GSE1993 dataset alone were used for molecular marker analysis. EGFR amplification and CDKN2A/2B loss was assessed by PCR analysis and p53 mutation was assessed by Single-Strand Conformation Polymorphism (SSCP) Analysis as described before [1]

Supplementary Results

The validation of the 16-gene signature in GSE4271 dataset (Phillips *et al* dataset)

The GSE4271 dataset comprised of 22 AA samples and 76 GBM samples [2]. Out of 16-genes of our signature set, the expression data was available for only 14-genes; the data for DCN and LGALS3 genes were not available. So, we used the expression data for the 14-genes and performed PAM analysis. Using PAM with a 10-fold cross validation **(Supplementary Figure S5A)** the 14 genes of the 16-gene signature was able to predict 12 AA samples out of 22 correctly with an error rate of 0.45. Similarly, among 76 GBM samples used, our 16-gene signature predicted 68 samples correctly as GBM with an error rate of 0.1 **(Supplementary Figure S5A)**. Thus, the 16 gene expression signature could discriminate GBM from AAs with an overall diagnostic accuracy of 81.6% (**Table 2**). The sensitivity for AA is 54.5%, whereas for GBM, it is 89.4%; the specificity for AA is 89.4%, whereas for GBM, it is 54.5% (**Table 2**). While we do not know the exact reason for the low accuracy of 16 gene signature in classifying high grade glioma from GSE4271 (Phillips dataset), one possible reason could be because of the missing data for 2 genes of the 16-gene signature.

Further to see if there is any difference in the clinical features of the authentic and discordant samples as per PAM of 14-genes, we looked at the average age and the survival of discordant and authentic AA and GBM samples. We included all the samples of this dataset: Authentic AAs (n=12), Authentic GBMs (n=68), Discordant AAs (n=10) and Discordant GBMs (n=8). As expected, the average age of Authentic AA (34 years) was significantly ($p < 0.0001$) lower than that of Authentic GBM (49.7 years) **(Supplementary Figure S5B)**. The Discordant AAs (41.4 years) were older in age as compared to the Authentic AAs (34 years) (though statistically not significant) whereas Discordant GBMs (38.6 years) were significantly ($p=0.04$)

younger in age as compared to the Authentic GBMs (49.7 years) **(Supplementary Figure S5B)**. In addition, the average age of Discordant AAs was similar to Authentic GBM (p=0.06) whereas the average age of Discordant GBMs was similar to Authentic AA (p=0.36).

With respect to patient survival, as expected, the survival length of Authentic AA (median survival=61 months/ 5.1 years) was significantly (p < 0.004) higher than that of Authentic GBM (median survival=15.5 months/ 1.3 years). Further analysis revealed that the median survival of Discordant AAs (median survival=27 months/ 2.25 years) was significantly (p=0.02) lower than that of Authentic AAs (median survival=61 months/ 5.1 years) **(Supplementary Figure S5C)**. Similarly, the Discordant GBMs (median survival=51 months/ 4.2 years) had statistically significant (p=0.03) better survival as compared to the Authentic GBMs (median survival=15.5 months/ 1.3 years) **(Supplementary Figure S5C)**. This suggests that though the 14-genes of the signature are inadequate for the accurate classification of AA and GBM samples, there is a trend of discordant AA and GBMs belonging to the other group.

The validation of Petalidis gene signature in TCGA dataset to see its potential in classifying AA and GBM

We performed analysis to check the potentiality of the Petalidis gene signature in the classification of AA and GBM samples of the TCGA dataset. Out of 59 genes of the Petalidis signature, the data was available for 54 genes in the TCGA dataset. Thus, we have used the 54 genes for the PAM analysis. The PAM analysis revealed that the 55 genes were able to classify the AA and GBM samples with 100% accuracy in the TCGA dataset at threshold 0.0 **(Supplementary Figure S6)**.

The validation of Phillips gene signature in our dataset to see its potential in classifying AA and GBM

We performed additional analysis wherein the ability of Phillips gene signature to classify AAs from GBMs in our dataset was addressed. The results of these analyses are described subsequently. In Phillips *et al* paper [2], a set of 8 genes are described as a marker for 3 prognostic subclasses of high grade glioma: Olig2, DLL3 and BCN as Proneural markers, PCNA and TOP2A as Proliferative markers and CHI3L1/YKL40, CD44 and VEGF as Mesenchymal markers.

Out of the 8 genes of the signature, we used the expression data (obtained from our dataset) of Olig2, DLL3 and BCAN as markers of Proneural subclass while that of TOP2A and CHI3L1 as markers for other two subclasses. From the PAM analysis (**Supplementary Figure S7A**), it is clear that these 5 genes were not adequate for the classification of AA and GBM samples: the classification sensitivity for AA being only 44% (22 of 50 AAs are rightly classified) and the specificity for AA was 99%. However, the classification sensitivity for GBM was 99% (131 of 132 GBMs are rightly classified) and the specificity for GBM was 44% (**Supplementary Figure S7B**). Thus it appears that Phillips *et al* gene signature cannot be used classification of AAs from GBMs.

The inadequacy of the Phillips gene signature to accurately classify AA and GBM in our dataset could be because of the lack of expression data for 3 genes. To rule out this possibility, we verified the ability of Phillips gene set to classify AAs from GBMs in Phillips dataset itself and also other datasets, TCGA and GSE4422. In this analysis, we used all the 8 genes as the expression data was available for all the genes.

i) Classification analysis of Phillips gene set in Phillips dataset.

In this dataset, the sensitivity of prediction of AA was only 50% (12 out of 24 AAs were rightly identified) and that for GBM was 93.4% (71 of 76 GBMs are rightly classified) **(Supplementary Figure S8A and S8B)**. The specificity of the signature for AA was 93.4% and for GBM it was 50%. Thus it appears that the 8-gene signature of Phillips was not able to accurately classify the samples of Phillips dataset into AA and GBM. The low classification sensitivity for AAs reiterates the fact that the Phillips gene signature is meant for identifying the prognostic classes, but not meant for distinguishing AA and GBM.

ii) Classification analysis of Phillips gene set in GSE4422 dataset.

We applied Phillips 8-gene signature in GSE4422 **(Supplementary Figure S9A and S9B)**. It is interesting to note that, in this dataset, the Phillips-gene signature could not predict any of the AA samples as AA suggesting that the prediction sensitivity was 0% for AA samples of this dataset and that for GBM was 100%. The specificity of the signature was 100% for AA and 0% for GBM.

iii) Classification analysis of Phillips gene set in TCGA dataset.

Next, we applied Phillips 8-gene signature to TCGA dataset **(Supplementary Figure S10A and S10B)**. In this dataset, the classification accuracy for AA was 85% (23 out of 27 samples were rightly classified) and the specificity for AA was 100%. In case of GBMs, the sensitivity was 100% and the specificity of prediction was 85%.

Overall, our analyses of Phillips gene-signature across various datasets suggest that the Phillips gene signature fails to consistently predict AA and GBM samples with high accuracy.

The sensitivity of the signature for AA varies greatly across datasets: 0% in GSE4422 dataset; 44% in our dataset, 50% in Phillips dataset and 85% in TCGA dataset. In particular, the Phillips gene signature fails to predict AA samples accurately, thus compromising the sensitivity for AA prediction and specificity for GBM prediction. A possible reason for the inability of Phillips gene signature to classify AAs from GBMs is that the signature was not developed for this purpose.

Reference

1. van den Boom J, Wolter M, Kuick R, Misek DE, Youkilis AS, et al. (2003) Characterization of gene expression profiles associated with glioma progression using oligonucleotide-based microarray analysis and real-time reverse transcription-polymerase chain reaction. Am J Pathol 163: 1033-1043.
2. Phillips HS, Kharbanda S, Chen R, Forrest WF, Soriano RH, et al. (2006) Molecular subclasses of high-grade glioma predict prognosis, delineate a pattern of disease progression, and resemble stages in neurogenesis. Cancer Cell 9: 157-173.

Supplementary Tables

Supplementary table S1. Primers used for RT-qPCR

Gene Symbol	Forward primer : Sequence (5'-3')	Reverse primer : Sequence (5'-3')
**18S**	TAACAGGTCTGTGATGCCCT	TCAAGTTCGACCGTCTTCTC
**ADD3**	GCCAAGGCGTGATTACCACT	TGCAGGATCTGAGTAACTCGTTT
**AEBP1**	TGCTCTGTGGCCCCTGTCTACA	CACCTTCATGAGCTGGCGCAT
**AGPAT1**	GACGCAACGTCGAGAACATGAA	CTCCATCATCCCAAGCAGATCG
**AGT**	CCCCAGTCTGAGATGGCTC	GAGGTGGAAGGGGTGTATGT
**AQP1**	TTAACCCTGCTCGGTCCTTTG	AGTCGTAGATGAGTACAGCCAG
**ARC**	CTGTCCCAGATCCAGAATCACATGA	TTGCGCCACAGGAACTGGTC
**ARHC**	CCTGCCTCCTCATCGTCTTC	AGCACATGAGGATGACATCAGTG
**ASCL1**	AAGCTCTGCCAAGATGGAGA	GGCAAAGAAACAGGCTGC
**ATP5G1**	CCAGACGGGAGTTCCAGAC	GACGGGTTCCTGGCATAGC
**B2M**	AGGCTATCCAGCGTACTCCAA	AATGCGGCATCTTCAAACC
**BCAN**	GCCTGACAGCTACTTCCTGTCTGGA	CAGGACACCAGCCCCATCTTG
**BHC80**	AAAGTGGAAATTCAGGTTCACCA	TCAGGTTCTTCCGTAGCTGTT
**BMP2**	ACCCGCTGTCTTCTAGCGT	CTCAGGACCTCGTCAGAGGG
**C1QB**	AGGGAACCTGTGCGTGAAC	CTCCATGCCCAGTAGTGAGTT
**C5orf18**	GGCTGACCTACTGGGTAGTGT	GGATGATGCGCTTGTAGAGC
**C6ORF66**	AGCGGAACGGGAAATCAGC	GCAGCTTTTACCTGCAAGGAA
**CACNG4**	CGAAGGGATCTATAAAGGGCAC	ACTGAGGACGATGTTGTTCTTG
**CALCRL**	CAGCAAGCAACAGAACATGGA	GTGATGCAATAGACAATCCGTGT
**CBX3**	TAGATCGACGTGTAGTGAATGGG	TGTCTGTGGCACCAATTATTCTT
**CCL2**	CGCTCAGCCAGATGCAATCAAT	GCTGGTGATTCTTCTATAGCTCGCG
**CD99L2**	TGGTAGCAAGAGTGACCGATGTC	CAAGCGAATGTCTCTTTCCCCTC
**CDC2**	AGGTCAAGTGGTAGCCATGAA	ACAAAACACAATCCCCTGTAGG
**CDKN3**	TCAGTTTCTCGGTTTATGTGCTC	TTTTGACAGTTCCCCTCTGGT
**CENPF**	ACAACTGTCACTTGAGCTGGA	GTGGGTTTCAATGCACCAGTC
**CHI3L1**	GTGAAGGCGTCTCAAACAGG	TCAGGCTGGCACCTTTAGTG
**CITED1**	GACCCCTGCAAAACTGGGAC	ACCAGCGACATCAGCACTTC
**CLU**	ACGAAGAGCGCAAGACACTG	ATGGTCTCATTGCACACTCCT
**CNR1**	AAGGTGACATGGCATCCAAAT	AGGACGAGAGAGACTTGTTGTAA
**COL4A2**	TTGGCGGGTGTGAAGAAGTTT	CCTTGTCTCCTTTACGTCCCTG
**COL6A1**	ACACCGACTGCGCTATCAAG	CACCGAGAAGACTTTGACGC
**COL6A3**	TGGTCCAGTTCAACGGAAACC	CACTACGATAACCTGAGGGACT
**CPE**	TGCAACGAATACCAGAAGGGG	CCAGTCCTTGAGTTCACCAGG
**CRB1**	GCAGATGACTTGATCTCCGAC	CCCTGAGTTGCCCTTTTGTTG
**CRTAC1**	CTGGCGCTCAGAGATGTGG	CCATTCTCATTGTCGCAGAAGAT

**CRYAB**	GTTCTTCGGAGAGCACCTGTT	GAGAGTCCAGTGTCAAACCAG
**CSDC2**	AAGCGGACCAGGACCTATTCA	CCCCTCGATGTCAGACACA
**CX43**	TGTCCTTAAGTCCCTGCTAA	GTAGCTGAGGAATGATGAAAAAG
**DBI**	TTAAGACCAAGCCATCGGATG	CCTTTCAGCTCATTCCAGGC
**DCN**	AGTTGGAACGACTTTATCTGTCC	GTGCCCAGTTCTATGACAATCA
**DCX**	TGGAGATGCTAACCTTGGGTA	TCACACATGCCCACATGACTA
**DDR1**	GGATGGAGCAACCACAGCTTCTC	GCATGTTGTTACAGTGGACCTGCAT
**DiRas2**	CTGGTGTTGAGGTTTGTGAAAGG	CCGTCGTGTCGGTGATCTG
**DLL1**	AGCTACACTTGCTCTTGCCGGC	CGTGCAGCTCCCTCCGTTCTTA
**DLL3**	GGTGAATGCCGATGCCTAGAG	GTGGTAGCAGAGGACGGGCC
**DLL4**	GCTGTGTAACGAATGCATCCCC	GAGATCTTGGTCACAAAACAGGCCT
**DPP6**	AAGCCCTACCACTATCCCAAGGC	TCAGGCGGCATCATCTCCA
**DPYSL5**	GACGCTTATGAGAAGTGCCGA	CTCACCAGTGTCTCCATTTCTG
**EBPL**	GGTCTCTGGCATTGTTCCTCA	AACAGGTAAAGCCAACAGTACAG
**EDNRB**	CCATTTGGAGCTGAGATGTGTAAGC	CCAAGAAGCAACAGCTCGATATCTG
**EGFR**	GCGTTCGGCACGGTGTATAA	GGCTTTCGGAGATGTTGCTTC
**EML4**	TCGCCTGTCAGCTCTTGAGT	AGGCCACATGATCTTCAGAGATT
**ERBB2**	GACGAATTCTGCACAATGGCG	CGAAGCAGAGGTGGGTGTTATG
**FABP7**	CTCTCAGCACATTCAAGAACACG	GCGAACAGCAACCACATCAC
**FN1**	GCAGTAACCACTATTCCTGCAC	TCCTGATACAACCACGGATGAG
**FSTL1**	CAACCACTGTGAACTGCATCG	CCTTTAGAGAACCAGCCATCTG
**FTL**	ACGAGCGTCTCCTGAAGATG	CCCAGGGCATGAAGATCCAAA
**FXYD6**	ACCCTGAGGATTGGGGGAC	CATTGGCGGTGATGAGGTT
**GADD45A**	GAGAGCAGAAGACCGAAAGGA	CACAACACCACGTTATCGGG
**GDF8**	GGAAACAGCTCCTAACATCAGC	TGTCATCCCTCTGGACATCATAC
**GLCCI1**	GCAGCTCACCTGAGAGACG	AGGAGGTTCGCCTTATTGTACTA
**GLUD1**	CTGGCTTGGCATACACAATG	GCTGTTCTCAGGTCCAATCC
**GPM6B**	AGACCTGCAAACTTGTGCCA	CCCACAGCCGCAGAATAAG
**GRIA2**	TGTTGGAGTCCACGATGAACG	GCAAGATTTACTGGGGTTCCTAA
**GYG2**	AGGTCACCTGAAGCAGTTTAGCC	CCTGAAATATCCTACGAGCATCG
**HDAC4**	CACGAGCACATCAAGCAACAA	CAGTGGTTCAGATTCCGGTGG
**HES1**	GAGAGGCGGCTAAGGTGTTTG	CTGGTGTAGACGGGGATGAC
**HES2**	TGGACAGGTTGAAGCTCTTGG	CCTTTTATTCCCTGAGCCGAG
**HES6**	CCTTGGTGACCAATGCCAG	CCTGCAAGCCATCCATCAG
**HEY2**	GGCGTCGGGATCGGATAAATA	AAGTAGCCTTTACCCCCTGTT
**HLA-B**	TTCGTGAGGTTCGACAGCG	TGTAGTAGCCGCGCAGGTTC
**HMGN2**	TCCCTGACTTTGACACACATGG	GATTGCCTGACACACTGGTAACC
**IGF2**	CCTCCAGTTCGTCTGTGGG	CACGTCCCTCTCGGACTTG
**IGFBP1**	GAGAGCACGGAGATAA CTGAGG	TTGGTGACATGGAGAGCCTTCG
**IGFBP10**	TGTGGAACTGGTATCTCCACACGAG	ACTGATGTTTACAGTTGGGCTG
**IGFBP10**	ACCGCTCTGAAGGGGATCT	TTTTCAGGCTGCTGTACACTGGCT

**IGFBP2**	GACAATGGCGATGACCACTCA	GCTCCTTCATACCCGACTTGA
**IGFBP3**	AGAGCACAGATACCCAGAACT	TGAGGAACTTCAGGTGATTCAGT
**IGFBP4**	CCCACGAGGACCTCTACATCA	CGTCTTCCGGTCCACACAC
**IGFBP5**	CGGGGTTTGCCTCAACGAA	TCTTGGGGGAGTAGGTCTCCT
**IGFBP6**	CTGACCATGACCCCCCACA	CACTCCTGCCCCTCCCTCC
**IGFBP7**	GGTCCTTCCATAGTGACGCC	TCTGAATGGCCAGGTTGTCC
**IGFBP8**	CAGCATGGACGTTCGTCTG	CCAACCACGGTTTGGTCCTT
**IGFBP9**	CAATAGGAACCGTCAATGTGAGA	CTTGGTGCGGAGACACTTTTT
**IGFBPL1**	GTCACGAAGTCCCCTGAGGG	CCGTGGCCTCATGGTCAGA
**ITPKB**	GACTTCAGGAACCGACTCCG	TTCTCCATCCTTACAAAAGCGG
**JAG1**	GTCCATGCAGAACGTGAACG	GCGGGACTGATACTCCTTGA
**JAG2**	AGCTGGACGCCAATGAGTG	GTCGTTGACGTTGATATGGCA
**LAMB1**	ACAAGCCCGAACCCTACTGTA	GACCACATTTTCAATGAGATGGC
**LFNG**	GACCACCAAAAAGTTCCACCG	GGCCGAGCAGTTTGTGATGA
**LGALS1**	CTCCTGACGCTAAGAGCTTCG	CCAGGCTGGAAGGGAAAGAC
**LGALS3**	TGCTGATAACAATTCTGGGCAC	TGAAGCGTGGGTTAAAGTGGA
**LIF**	GTACCGCATAGTCGTGTACCT	CACAGCACGTTGCTAAGGAG
**LOX**	CAGGGTGCTGCTCAGATTTCC	GGTAATGTTGATGACAACTGTGC
**MAL2**	TTGCCTCCTCCAATGTTCCTC	CAGTTAGCATCAATTTGAGCCAC
**MBP**	CTCTGGATCACCCATGGCTAG	GAAATTGCCGGTAGGCTGC
**MCF2**	GGAGAGGCAAGATGAGGTTCA	CTCAGGGGTTAATTGCTTGTCA
**MCM2**	ATGGCGGAATCATCGGAATCC	GGTGAGGGCATCAGTACGC
**MET**	TGGTGCAGAGGAGCAATGG	CATTCTGGATGGGTGTTTCCG
**MGP**	AGATGGAGAGCTAAAGTCCAAGA	GTAGCGTTCGCAAAGTCTGTA
**MMP2**	CCGTCGCCCATCATCAAGTT	CTGTCTGGGGCAGTCCAAAG
**MMP9**	CATTTCGACGATGACGAGTTGT	CGGGTGTAGAGTCTCTCGC
**NCALD**	GACTTGCTGGAAAGCACAGAC	ATTTGGAAGCATCCCCATAAGG
**NOTCH1**	GAGGCGTGGCAGACTATGC	CTTGTACTCCGTCAGCGTGA
**NOTCH2**	CTGTGAGTGTCTGAAGGGTTATG	GGCACTGGAAACGATTGACTTT
**NOTCH3**	CGTGGCTTCTTTCTACTGTGC	CGTTCACCGGATTTGTGTCAC
**NOTCH4**	CTGGGTGTCAATGGAGAGGGA	GGGTGAGACGTGCCAGTTTC
**NSEP1**	AGCCACCTCAACGTCGGTA	TGGATCGGCTGCTTTTGTCTC
**NTRK2**	GATAAGCTGGACTCGGCACG	GGACGACATCCCTAGCAGCC
**NUDT10**	CTCCTGGGCGTCTTCGAAC	GCTAACCGAATCTTCCCAATCC
**OCIL**	GCTGCTTTAAGCGCAATAAGAG	AACCTGAGCAAGATCAGCATC
**OLFM1**	AGACCATGTGTTCACGGGATG	AGGTGTTGCTTATGACTCTCCT
**OLIG1**	CGGACGCCAAGGAGGAGC	TATCTTGGAGAGCTTGCGGCC
**OLIG2**	GGACAAGCTAGGAGGCAGTG	ATGGCGATGTTGAGGTCGTG
**PACSIN1**	CACCGTCTATGCAACGACCTG	GCCTCTGTCATTATGGCACCC
**PBEF1**	ATTGCCTTCGGTTCTGGTGG	CGGCCCTTTTTGGACCTTTTG
**PCDHGA11**	TCAGACCTCTCGCTGTACTTG	CCGTCCACGCCTACAAAATGT

**PHLDA1**	GGAGATCGACTTTCGGTGCC	CTTCTGCCGCGTGGATTTG
**PHLPP**	ACTGGGATTTGGGGAGCTG	CGTCTTGTCCATCGGTTCACT
**PLAT**	ACTGCCGGAATCCTGATGG	TGTGCTTGGCAAAGATGGC
**PLEKHB1**	AAAGATCGGCCCAGAGTGC	GGAGTTTGCCTCCAGCAGTG
**PLEKHH2**	CTCCGAAACCCTTATCACCATTC	TGAACAACGCAAATCCAGACTG
**PTBP1**	GAGCCCCGGTCCTATCTTAGAG	GCAGGCAAAACACAGAAATGAG
**PTPN13**	ACTCCAGCAGTCAGGATCATC	ATATCTCCAGGCTTAGGAGGTG
**PTPRO**	TTCCATACAACTGGCGTAGGA	CCAAAGACATGAGGGTAGCAG
**PTPRZ1**	GCATGTTTCCAGGGTCCCTT	GTCACCAGATGCTTGAGAGAC
**PTTG1**	TGATCCTTGACGAGGAGAGAG	GGTGGCAATTCAACATCCAGG
**QKI**	CTCTGTCCTGTGGTCCCGTG	AGATCTTGCGCTGAAAGTGCTG
**RAB13**	ATAACTACTGCCTACTACCGTGG	CCATGTCACATTTGTTCCCCAG
**RAB26**	GTCTGCTGGTGCGATTCAAG	GCATGGGTAACACTGCGGA
**RAN**	AGGACAGGAAAGTGAAGGCG	TGGCAACAAATTCCAAGTTAGG
**RFNG**	TGTGTATGGTGATGGTGAGTCGG	CAGCATTGAAGCATGGCATGAG
**RGS4**	CAAGCCGGAACATGCTAGAG	CGGGTTGACCAAATCAAGATAGA
**RND2**	GCGAGCTTTGAGATCGACAAG	CAGTGTTTCTGGTCGGCTAAT
**RPL35A**	GGGTACAGCATCACTCGGA	ACGCCCGAGATGAAACAG
**RPLP1**	ATTCTGCACGACGATGAGGTG	GATGAGGCTCCCAATGTTGAC
**RPS19**	AAAGAGCTTGCTCCCTACGAT	CATGACGCCGTTTCTCTGAC
**RPS3**	TGCGAGGTTGTGGTGTCTG	ACAGCAGTGTCAACGTAGTAGT
**RTN3**	GGACGTAGACATTACTCTGTCCT	CACCAACATAGGTCATCAGCC
**S100A4**	GATGAGCAACTTGGACAGCAA	CTGGGCTGCTTATCTGGGAAG
**S100A6**	GGCTGATGGAAGACTTGGACC	TTTGACTCAGAGAGGACCCCC
**SATB1**	ACAGGTGCAAAAATGCAGGGA	GCGTTTTCATAATGTTCCACCAC
**SEMA4D**	TGTGACCACCTGAACTTAACATC	TCTCCATCAACCATGACGGAT
**SHC3**	ACCTCCAGTTTGCGGGAATG	CGCATGTGGTGATTCGCTATG
**SLC1A3**	GAATGGCGGCGCTAGATAGTA	GTGCATGTTTTCCTTTGTGCC
**SMC5L1**	TGCCATTTGCCTTGGTTTAGC	CACGGGTGATTACAAGATTTCCA
**SNCA**	ATGTAGGCTCCAAAACCAAGG	CCTCCAACATTTGTCACTTGCT
**SNCB**	ATGAAGGGCCTGTCCATGG	TTCCGACGTAGAGGACGCC
**SOD2**	AACCTCAGCCCTAACGGTG	AGCAGCAATTTGTAAGTGTCCC
**SPARC**	CGAGACCTGTGACCTGGACAATG	TCCGGTACTGTGGAAGGAGTGG
**SPARCL1**	GCACCTGACAACACTGCAATC	TTTCAGCCTTATGGTGGGAATC
**SYT1**	GGTTGGCTGTTTCCCAGTAAAAC	TTTTAAGAAGTACGGACCATCGG
**SYT5**	TGTTTCTGTCTCTACCGGAAGA	GCACCTTGTCTATGTAACTCTGG
**TFAP2A**	AGGTCAATCTCCCTACACGAG	GGAGTAAGGATCTTGCGACTGG
**TFAP2B**	GCCCCCTGAATGCCTCAAT	AGTAACGTGACATTTGCTGCTT
**TFAP2C**	CGGAGCGGTCTTGACACTC	CAAGCGGTAAATCCAAAATCGG
**TFAP2D**	TCCTTCCATTACGAGTTTCAGC	GCAGGTTAATAAAGTCGGTGGG
**TFAP2E**	GGTGTTTGCGGGAACGGTTA	CTCTCCTTCCACTAGCGAAGT

**TGFBR1**	CCTAGGATGCTCACCTTCCAAG	AGGTTCCAAGAAACAGCTGGAG
**THBS1**	CCTGACCGTCCAAGGAAAGC	CCTTTGCGATGCGGAGTCT
**THBS2**	ACAAAGACACGACCTTCGACC	GACTTGCCGTCCTGCTTGA
**THBS4**	TGCTGCCAGTCCTGACAGA	GTTTAAGCGTCCCATCACAGTA
**TIAM1**	GATCCACAGGAACTCCGAAGT	GCTCCCGAAGTCTTCTAGGGT
**TIMP1**	CACCAGAGAACCCACCATGG	GCTGGTATAAGGTGGTCTGGTTG
**TOP2A**	CTCGCTTGTCATTCCGTTTGA	CAACGAAAGTTACTTGGGCTTCC
**TRIM9**	TGGTTCATGCACAACAACTCG	CCCTCCACGTTATCAAATGCT
**UEST 275430**	ATGAATTGTCGGTTGTCCTGC	AATTTTCCCCCACCTGTTCTG
**UEST 39152**	GACATTTACGCCACAACCCAC	AGACCCTCAGATTCCCCACC
**VEGFA**	CAACATCACCATGCAGATTATGC	TCGGCTTGTCACATTTTTCTTGT
**VIM**	CAGGAACAGCATGTCCAAATCG	TGTACCATTCTTCTGCCTCCTGC
**VPS13D**	CGATGCCTCTTGCTGGAATG	TCTCTCGGATGTCTCTCTGCAG
**VSNL1**	ATGGGGAAGCAGAATAGCAAAC	CTCCCACTTGGACAGTCCTTG
**YES1**	TTTTTGAGACGGAGTCTCGCTC	CACGGTGAAACTGCGTCTCTAC
**ZNF224**	TTCAGAAGCAGGAACACATCAAG	GGATGGTTTATATCCAATTGCCC
**ZNF35**	GCCTGTAACGACTGTGGCAA	ACAATAAGGCAAGAGAGCTGACT

Supplementary Table S2. List of genes selected for expression analysis by PCR array.

S.No.	Symbol	UGRepAcc	GENE FULL NAME	Author
1	DiRas2	NM_017594	DIRAS family, GTP-binding RAS-like 2	Somasundaram et al, 2005
2	MMP2	NM_001127891	matrix metallopeptidase 2	Somasundaram et al, 2005
3	PTBP1	NM_002819.3	polypyrimidine tract binding protein 1	Somasundaram et al, 2005
4	TGFBR1	NM_001130916	transforming growth factor, beta receptor 1	Somasundaram et al, 2005
5	UEST	NM_002819.3	Homo sapiens cDNA clone nav32d02 5-, mRNA sequence	Somasundaram et al, 2005
6	YES1	NM_005433	Yamaguchi sarcoma viral oncogene homolog 1	Somasundaram et al, 2005
7	DCN	NM_001920	decorin	Somasundaram et al, 2005
8	LGALS3	NM_001177388	lectin, galactoside-binding, soluble, 3	Somasundaram et al, 2005
9	VEGFA	NM_001025366	vascular endothelial growth factor A	Sophie Godard et al 2003
10	FN1	NM_002026	fibronectin 1	Tso et al, 2006
11	MMP9	NM_004994	matrix metallopeptidase 9	Tso et al 2006
12	MGP	NM_000900	matrix Gla protein	Somasundaram et al, 2005
13	THBS2	NM_003247	thrombospondin 2	Somasundaram et al, 2005
14	TFAP2C	NM_003222	transcription factor AP-2 gamma	Somasundaram et al, 2005
15	TFAP2D	NM_172238	transcription factor AP-2 delta	Somasundaram et al, 2005
16	TFAP2E	NM_178548	transcription factor AP-2 epsilon	Somasundaram et al, 2005
17	TFAP2B	NM_003221	transcription factor AP-2 beta	Somasundaram et al, 2005
18	ADD3	NM_001121	adducin 3	Somasundaram et al, 2005
19	AEBP1	NM_001129	AE binding protein 1	Reddy et al 2008
20	AGT	NM_000029	angiotensinogen	Somasundaram et al, 2005
21	AQUA	NM_198098.1	Homo sapiens aquaporin 1 (channel-forming integral protein, 28kDa)	Somasundaram et al, 2005
22	ARC	BAA19667	activity-regulated cytoskeleton-associated protein	Somasundaram et al, 2005
23	ARHC	NM_175744.3	ras homolog gene family, member C	Somasundaram et al, 2005
24	ASCL1	NM_004316	achaete-scute complex homolog 1	Somasundaram et al, 2005
25	B2M	NM_004048	beta-2-microglobulin	Somasundaram et al, 2005
26	BCAN	NM_021948	brevican	Somasundaram et al, 2005
27	BHC80	NM_001101802	PHD finger protein 21A	Somasundaram et al, 2005
28	BMP2	NM_001200	bone morphogenetic protein 2	Somasundaram et al, 2005
29	C1QB	NM_000491	complement component 1, q subcomponent, B chain	Somasundaram et al, 2005
30	C5ORF18	NM_005669	polyposis locus protein 1; deleted in polyposis 1; polyposis coli region hypothetical protein DP1 [Homo sapiens].	Somasundaram et al, 2005
31	C6ORF66	NM_014165	NADH dehydrogenase (ubiquinone) 1 alpha subcomplex, assembly factor 4	Somasundaram et al, 2005
32	CACNG4	NM_014405	calcium channel, voltage-dependent, gamma subunit 4	Somasundaram et al, 2005
33	CALCRL	NM_005795	calcitonin receptor-like	Somasundaram et al, 2005
34	CBX3	NM_007276	chromobox homolog 3	Somasundaram et al, 2005
35	CCL2	NM_002982	chemokine (C-C motif) ligand 2	Somasundaram et al, 2005
36	CD99L2	NM_001184808	CD99 molecule-like 2	Somasundaram et al, 2005
37	CDC2	NM_001786	cell division cycle 2, G1 to S and G2 to M	Tso et al 2006

38	CDKN3	NM_001130851	cyclin-dependent kinase inhibitor 3	Ladha et al
39	CENPF	NM_016343	centromere protein F	Ladha et al
40	CHI3L1	NM_001276	chitinase 3-like 1	Tso et al 2006
41	CITED1	NM_001144885	Cbp/p300-interacting transactivator, with Glu/Asp-rich carboxy-terminal domain, 1	Somasundaram et al, 2005
42	CLU	NM_203339	clusterin	Somasundaram et al, 2005
43	CNR1	NM_001160226	cannabinoid receptor 1	Somasundaram et al, 2005
44	COL4A2	NM_001846.1	collagen, type IV	Somasundaram et al, 2005
45	COL6A1	NM_001848	collagen, type VI, alpha 1	Somasundaram et al, 2005
46	COL6A3	NM_004369	collagen, type VI, alpha 3	Somasundaram et al, 2005
47	CPE	NM_001873	carboxypeptidase E	Somasundaram et al, 2005
48	CRB1	NM_001193640	crumbs homolog 1	Somasundaram et al, 2005
49	CRTAC1	NM_001206528	cartilage acidic protein 1	Somasundaram et al, 2005
50	CRYAB	NM_001885	crystallin, alpha B	Somasundaram et al, 2005
51	CSDC2	NM_014460	cold shock domain containing C2, RNA binding	Somasundaram et al, 2005
52	CX43(MAG17)	NM_000165.2	connexin 43	Somasundaram et al, 2005
53	DBI	NM_001079862	diazepam binding inhibitor	Somasundaram et al, 2005
54	DCX	NM_000555	doublecortin	Somasundaram et al, 2005
55	DDR1	NM_001202521	discoidin domain receptor tyrosine kinase 1	Somasundaram et al, 2005
56	DLL1	NM_005618	delta-like 1 protein precursor; delta homolog [Homo sapiens].	Somasundaram et al, 2005
57	DLL3	NM_016941	delta-like 3 protein precursor; delta homolog [Homo sapiens].	Somasundaram et al, 2005
58	DLL4	NM_019074	delta-like 4 protein precursor; delta homolog [Homo sapiens].	Somasundaram et al, 2005
59	DPP6	NM_001039350	dipeptidyl-peptidase 6	Somasundaram et al, 2005
60	DPYSL5	NM_020134	dihydropyrimidinase-like 5	Somasundaram et al, 2005
61	EBPL	NM_032565	emopamil binding protein-like	Somasundaram et al, 2005
62	EDNRB	NM_000115	Endothelin receptor type B	Somasundaram et al, 2005
63	EGFR	NM_005228	epidermal growth factor receptor	Somasundaram et al, 2005
64	EML4	NM_001145076	echinoderm microtubule associated protein like 4	Somasundaram et al, 2005
65	ERBB2	NM_001005862	v-erb-b2 erythroblastic leukemia viral oncogene homolog 2	Somasundaram et al, 2005
66	FABP7	NM_001446	fatty acid binding protein 7, brain [Homo sapiens]	Somasundaram et al, 2005
67	FLJ10619	AI378412	Homo sapiens vacuolar protein sorting 13D (yeast) (VPS13D)	Somasundaram et al, 2005
68	FSTL1	NM_007085	follistatin-like 1	Somasundaram et al, 2005
69	FTL	NM_000146	ferritin, light polypeptide	Somasundaram et al, 2005
70	FXYD6	NM_022003	FXYD domain containing ion transport regulator 6	Somasundaram et al, 2005
71	GADD45A	NM_001199741	Homo sapiens growth arrest and DNA-damage-inducible, alpha (GADD45A), mRNA	Somasundaram et al, 2005
72	GDF8	NM_005259	Growth differentiation factor 8	Somasundaram et al, 2005
73	GLCCI1	BC050291	glucocorticoid induced transcript 1	Somasundaram et al, 2005
74	GLUD1	NM_005271	glutamate dehydrogenase 1	Somasundaram et al, 2005
75	GPM6B	NM_001001994	glycoprotein M6B	Somasundaram et al, 2005
76	GRIA2	NM_000826	glutamate receptor, ionotropic, AMPA 2	Somasundaram et al, 2005
77	GYG2	NM_003918.1	glycogenin 1	Somasundaram et al, 2005

78	HDAC4	NM_006037	Histone deacetylase 4	Somasundaram et al, 2005
79	HES1	NM_005524	hairy and enhancer of split 1,	Somasundaram et al, 2005
80	HES2	NM_019089	hairy and enhancer of split 2	Somasundaram et al, 2005
81	HES6	NM_001142853	hairy and enhancer of split 6	Somasundaram et al, 2005
82	HEY2	NM_012259	hairy/enhancer-of-split related with YRPW motif 2	Somasundaram et al, 2005
83	HLA-B	NM_005514	major histocompatibility complex, class I, B	Somasundaram et al, 2005
84	HMGN	NM_005517.2	High-mobility group nucleosomal binding domain 2	Somasundaram et al, 2005
85	IGF2	NM_000612	Insulin-like growth factor 2 (somatomedin A)	Somasundaram et al, 2005
86	IGFBP1	NM_000596	insulin-like growth factor binding protein 1 [Homo sapiens].	Somasundaram et al, 2005
87	IGFBP10	NM_001554	insulin-like growth factor binding protein 10 [Homo sapiens].	Somasundaram et al, 2005
88	IGFBP2	NM_000597	insulin-like growth factor binding protein 2 [Homo sapiens].	Tso et al 2006
89	IGFBP3	NM_000598	insulin-like growth factor binding protein 3 [Homo sapiens].	Somasundaram et al, 2005
90	IGFBP4	NM_001552	insulin-like growth factor binding protein 4 [Homo sapiens].	Somasundaram et al, 2005
91	IGFBP5	NM_000599	insulin-like growth factor binding protein 5 [Homo sapiens].	Somasundaram et al, 2005
92	IGFBP6	NM_002178	insulin-like growth factor binding protein 6 [Homo sapiens].	Somasundaram et al, 2005
93	IGFBP7	NM_001553	insulin-like growth factor binding protein 7 [Homo sapiens].	Somasundaram et al, 2005
94	IGFBP8	NM_001901	insulin-like growth factor binding protein 8 [Homo sapiens].	Somasundaram et al, 2005
95	IGFBP9	NM_002514	insulin-like growth factor binding protein 9 [Homo sapiens].	Somasundaram et al, 2005
96	IGFBPL1	NM_001007563	insulin-like growth factor binding protein-like 1	Somasundaram et al, 2005
97	IL1RL1	NM_003856.2	interleukin 1 receptor-like 1	Somasundaram et al, 2005
98	ITPKB	NM_002221	inositol 1,4,5-trisphosphate 3-kinase B	Somasundaram et al, 2005
99	JAG1	NM_000214.1	jagged 1	Somasundaram et al, 2005
100	JAG2	NM_002226	jagged 2	Somasundaram et al, 2005
101	KIAA2028	NM_172069.1	potassium channel tetramerisation domain containing 2	Somasundaram et al, 2005
102	KIAA0773	BC045611	KIAA0773 gene product [Homo sapiens].	Somasundaram et al, 2005
103	KIAA1102	NM_005109	KIAA1102 protein [Homo sapiens]	Somasundaram et al, 2005
104	LAMB1	NM_002291	Laminin, beta 1	Somasundaram et al, 2005
105	LFNG	NM_001040167	LFNG O-fucosylpeptide 3-beta-N-acetylglucosaminyltransferase	Somasundaram et al, 2005
106	LGALS1	NM_002305	lectin, galactoside-binding, soluble, 1 (galectin 1)	Somasundaram et al, 2005
107	LIF	NM_002309	leukemia inhibitory factor (cholinergic differentiation factor)	Somasundaram et al, 2005
108	LOX	NM_001178102	Lysyl oxidase	Somasundaram et al, 2005
109	MAL2	NM_052886	Mal, T-cell differentiation protein 2	Somasundaram et al, 2005
110	MBP	NM_001025081	myelin basic protein	Somasundaram et al, 2005
111	MCF2	NM_001099855	MCF.2 cell line derived transforming sequence	Somasundaram et al, 2005
112	MCM2	NM_004526	minichromosome maintenance complex component 2	Somasundaram et al, 2005
113	MET	NM_000245	Met proto-oncogene (hepatocyte growth factor receptor)	Tso et al 2006
114	MFNG	NM_001166343	MFNG O-fucosylpeptide 3-beta-N-acetylglucosaminyltransferase	Somasundaram et al, 2005
115	NCALD	NM_032041	neurocalcin delta [Homo sapiens]	Somasundaram et al, 2005
116	NOTCH2	NM_001200001	Notch homolog 2 [Homo sapiens].	Somasundaram et al, 2005
117	NOTCH3	NM_000435	Notch homolog 3 [Homo sapiens].	Somasundaram et al, 2005
118	NOTCH4	NM_004557	Homo sapiens Notch homolog 4 (Drosophila) (NOTCH4), mRNA	Somasundaram et al, 2005

119	NSEP1	NM_004559	DNA-binding protein B(actuaslly WANT TO DESIGN FOR 'nuclease sensitive element binding protein 1)	Somasundaram et al, 2005
120	NTRK2	NM_001007097	Neurotrophic tyrosine kinase, receptor, type 2	Somasundaram et al, 2005
121	NUDT10	NM_153183	Nudix (nucleoside diphosphate linked moiety X)-type motif 10	Somasundaram et al, 2005
122	OCIL	NM_013269	C-type lectin superfamily 2, member D	Somasundaram et al, 2005
123	OLFM1	NM_006334	olfactomedin related ER localized protein isoform 1; neuroblastoma protein; olfactomedin related ER localized protein; pancortin 1	Somasundaram et al, 2005
124	OLIG1	NM_138983	oligodendrocyte transcription factor 1	Somasundaram et al, 2005
125	OLIG2	NM_005806	oligodendrocyte transcription factor 2	Somasundaram et al, 2005
126	PACSIN1	NM_001199583	Protein kinase C and casein kinase substrate in neurons 1	Somasundaram et al, 2005
127	PBEF1	NM_005746.1	Homo sapiens pre-B-cell colony enhancing factor 1 (PBEF1), transcript variant 1, Mrna	Somasundaram et al, 2005
128	PCDHGA11	NM_018914	protocadherin gamma subfamily A, 11 isoform 1 precursor	Somasundaram et al, 2005
129	PHLDA1	NM_007350	pleckstrin homology-like domain, family A, member 1	Somasundaram et al, 2005
130	PHLPP	AB011178	PH domain and leucine rich repeat protein phosphatase	Somasundaram et al, 2005
131	PLAT	NM_000930	Homo sapiens plasminogen activator, tissue (PLAT)	Somasundaram et al, 2005
132	PLEKHB1	NM_001130033	pleckstrin homology domain containing, family B (evectins) member 1	Somasundaram et al, 2005
133	PTPN13	NM_006264	Protein tyrosine phosphatase, non-receptor type 13 (APO-1/CD95 (Fas)-associated phosphatase)	Somasundaram et al, 2005
134	PTPRO	NM_002848	Protein tyrosine phosphatase, receptor type, O	Somasundaram et al, 2005
135	PTPRZ1	NM_001206838	protein tyrosine phosphatase, receptor-type, Z polypeptide 1	Somasundaram et al, 2005
136	PTTG1	NM_004219	Pituitary tumor-transforming 1	Tso et al 2006
137	QKI	NM_006775	quaking homolog, KH domain RNA binding	Somasundaram et al, 2005
138	RAB13	NM_002870	Homo sapiens RAB13, member RAS oncogene family (RAB13)	Somasundaram et al, 2005
139	RAB26	NM_014353	RAB26 protein [Homo sapiens]	Somasundaram et al, 2005
140	RAN	NM_006325	RAN, member RAS oncogene family	Somasundaram et al, 2005
141	RFNG	NM_002917	Homo sapiens radical fringe homolog (Drosophila)	Somasundaram et al, 2005
142	RGS4	NM_001102445	Regulator of G-protein signalling 4	Somasundaram et al, 2005
143	RND2	NM_005440	Rho family GTPase 2	Somasundaram et al, 2005
144	RPLP1	NM_001003	ribosomal protein P1; 60S acidic ribosomal protein P1; acidic ribosomal phosphoprotein P1 [Homo sapiens	Somasundaram et al, 2005
145	RPS19	NM_001022	Homo sapiens ribosomal protein S19 (RPS19)	Somasundaram et al, 2005
146	RPS3	NM_001005	ribosomal protein S3; 40S ribosomal protein S3 [Homo sapiens].	Somasundaram et al, 2005
147	RTN3	NM_006054	reticulon 3 [Homo sapiens]	Somasundaram et al, 2005
148	S100A4	NM_002961	S100 calcium binding protein A4 (calcium protein, calvasculin, metastasin, murine placental homolog)	Somasundaram et al, 2005
149	S100A6	NM_014624	S100 calcium binding protein A6 (calcium protein, calvasculin, metastasin, murine placental homolog)	Somasundaram et al, 2005
150	SATB1	NM_001131010	Special AT-rich sequence binding protein 1 (binds to nuclear matrix/scaffold-associating DNA's)	Somasundaram et al, 2005
151	SEMA4D	NM_001142287	Homo sapiens sema domain, immunoglobulin domain (Ig), transmembrane domain (TM) and short cytoplasmic domain, (semaphorin) 4D SEMA4D), mRNA	Somasundaram et al, 2005
152	SHC3	NM_016848	SHC (Src homology 2 domain containing) transforming protein 3	Somasundaram et al, 2005
153	SLC1A3	NM_001166695	solute carrier family 1 (glial high affinity glutamate transporter), member 3 [Homo sapiens]	Somasundaram et al, 2005
154	SMC5L1	NM_015110	SMC5 protein (Homo sapiens)	Somasundaram et al, 2005
155	SNCA	NM_000345	Synuclein, alpha (non A4 component of amyloid precursor)	Somasundaram et al, 2005

156	SNCB	NM_001001502	Homo sapiens synuclein, beta (SNCB), transcript variant 1, mRNA	Somasundaram et al, 2005
157	SOD2	NM_000636	Homo sapiens superoxide dismutase 2, mitochondrial (SOD2), nuclear gene encoding mitochondrial protein, transcript variant 1,2, 3 mRNA	Tso et al 2006
158	SPARC	NM_003118	Homo sapiens secreted protein, acidic, cysteine-rich (osteonectin) (SPARC), mRNA	Somasundaram et al, 2005
159	SPARCL1	NM_001128310	SPARC-like 1; mast9; hevin [Homo sapiens].	Somasundaram et al, 2005
160	SYT1	NM_001135805	Human synaptotagmin I mRNA, 3' UTR	Somasundaram et al, 2005
161	SYT5	NM_003180	Synaptotagmin V	Somasundaram et al, 2005
162	TFAP2a	NM_001032280	transcription factor AP-2 alpha	Somasundaram et al, 2005
163	THBS1	NM_003246	Thrombospondin 1	Tso et al 2006
164	THBS4	NM_003248	thrombospondin 4 [Homo sapiens]	Somasundaram et al, 2005
165	TIAM1	NM_003253	Homo sapiens T-cell lymphoma invasion and metastasis 1 (TIAM1),	Somasundaram et al, 2005
166	TIMP1	NM_003254	Tissue inhibitor of metalloproteinase 1 (erythroid potentiating activity, collagenase inhibitor)	Tso et al 2006
167	TOP2A	NM_001067	Topoisomerase (DNA) II alpha 170kDa	Tso et al 2006
168	TRIM2	NM_001130067	tripartite motif-containing 2	Somasundaram et al, 2005
169	UEST_39152		Homo sapiens cDNA clone	Somasundaram et al, 2005
170	UEST_27543	AK123390.1	Homo sapiens cDNA FLJ41396 fis, clone BRCOC2019255	Somasundaram et al, 2005
171	UEST_f_218	T50536.1	yb30c07.r1 Stratagene fetal spleen (#937205) Homo sapiens cDNA	Somasundaram et al, 2005
172	VIM	NM_003380	vimentin	Sophie Godard et al 2003
173	VSNL1	NM_003385	Visinin-like 1	Somasundaram et al, 2005
174	ZNF224	NM_013398	Zinc finger protein 224/Homo sapiens zinc finger protein 2 mRNA, complete cds	Somasundaram et al, 2005
175	ZNF35	NM_003420	zinc finger protein 35 (clone HF.10); Zinc finger protein-35 (HF.10) [Homo sapiens].	Somasundaram et al, 2005
176	AGPAT*	NM_006411.2	acylglycerol-3-phosphate O-acyltransferase	Somasundaram et al, 2005
177	ATP5G1*	NM_005175.2	ATP synthase, H+ transporting, mitochondrial Fo complex, subunit C1 (subunit 9)	Somasundaram et al, 2005
178	GARS*	NM_002047.1	glycyl-tRNA synthetase (GARS)	Somasundaram et al, 2005
179	RPL35*	NM_007209	ribosomal protein L35	Somasundaram et al, 2005
180	18S rRNA*	X03205	18S ribosomal RNA	Somasundaram et al, 2005

Supplementary Table S3. Number of AA and GBM patient samples in training set, test set and three independent cohorts of patient samples (TCGA, GSE1993 and GSE4422).

Cohort	Dataset	AA	GBM	Reference
**Our cohort**	**Training set**	30	78	Reddy et al 2006
	**Test set**	20	54	Reddy et al 2006
**Validation sets**	**TCGA**	27*	152	Network TCGA, 2008
	**GSE1993**	19	39	Petalidis et al, 2008
	**GSE4422**	5	71	Freije et al, 2004
	**GSE4271**	22	76	Phillips et al, 2006

*27 grade III glioma samples consisted of 10 Anaplastic Astrocytoma, 9 Oligoastrocytoma and 8 Oligodendroglioma

Supplementary Table S4. Expression of 16 genes in AA (n=20) and GBM (n=54) samples of the test set.

Gene Name	Average of AA	Average of GBM	Fold change	P value*
**CDKN3**	0.3	2.1	1.8	4.1×10^{-5}
**CHI3L1**	1.3	5.1	3.8	1.4×10^{-7}
**COL4**	0.9	4.0	3.1	1.5×10^{-5}
**DCN**	0.6	2.2	1.6	8.0×10^{-3}
**DLL3**	6.3	2.4	-3.9	3.1×10^{-5}
**FABP7**	-2.4	0.5	2.9	1.0×10^{-6}
**IGFBP2**	1.9	5.2	3.3	2.1×10^{-10}
**IGFBP3**	1.0	3.7	2.7	7.1×10^{-4}
**LAMB1**	0.2	2.0	1.8	3.0×10^{-5}
**LGAL**	0.1	1.3	1.2	7.8×10^{-2}
**LGALS3**	0.1	2.7	2.6	6.6×10^{-8}
**PBEF1**	1.1	2.5	1.3	8.8×10^{-4}
**PLAT**	-0.7	2.1	2.8	1.1×10^{-6}
**PTTG1**	1.9	3.2	1.3	7.4×10^{-3}
**TIMP1**	-0.2	3.7	3.9	1.5×10^{-19}
**TOP2A**	5.6	8.1	2.5	2.6×10^{-6}

* P value from student's T-test

Supplementary Table S5. Expression of 16 genes in Grade III glioma (n=27) and GBM (n=152) samples of the TCGA dataset.

Gene Name	Average of Grade III	Average of GBM	Fold change (log)	P value*
CDKN3	-3.4	1.5	4.8	8×10^{-19}
CHI3L1	0.9	2.2	1.3	1×10^{-5}
COL4A2	-0.9	3.0	3.8	1×10^{-17}
DCN	-0.4	0.4	0.7	8×10^{-4}
DLL3	4.7	0.0	-4.7	1×10^{-59}
FABP7	3.6	0.8	-2.7	1×10^{-12}
IGFBP2	-2.7	4.3	7.0	3×10^{-25}
IGFBP3	-3.1	1.8	4.9	3×10^{-24}
LAMB1	-4.0	1.8	5.8	6×10^{-24}
LGALS1	-2.2	1.4	3.6	2×10^{-25}
LGALS3	-3.7	1.9	5.7	4×10^{-37}
PLAT	-1.6	1.7	3.2	1×10^{-15}
PTTG1	-3.3	1.8	5.2	2×10^{-20}
TOP2A	-3.3	2.9	6.3	1×10^{-17}
TIMP1	-3.2	2.8	6.0	9×10^{-30}

* P value from student's T-test

Supplementary Table S6. Expression of 16 genes in AA (n=19) and GBM (n=39) samples of GSE1993 dataset.

Gene Name	Average of AA	Average of GBM	Fold change	P value*
**CDKN3**	5.7	6.6	1.9	3.1×10^{-4}
**CHI3L1**	8.2	10.8	6.1	1.0×10^{-4}
**COL4A2**	7.3	9.1	3.5	1.1×10^{-5}
**DCN**	7.3	8.6	2.5	3.4×10^{-6}
**DLL3**	8.6	7.1	0.4	2.8×10^{-4}
**FABP7**	6.3	8.2	3.7	3.0×10^{-4}
**IGFBP2**	7.0	9.9	7.5	9.4×10^{-6}
**IGFBP3**	7.5	9.1	3.0	1.2×10^{-5}
**LAMB1**	4.5	5.4	1.9	1.4×10^{-5}
**LGALS1**	9.2	10.9	3.2	4.9×10^{-6}
**LGALS3**	8.0	9.8	3.5	6.5×10^{-6}
**NAMPT**	6.8	7.9	2.1	9.9×10^{-4}
**PLAT**	5.7	7.3	3.0	2.6×10^{-5}
**PTTG1**	7.8	9.2	2.6	2.3×10^{-5}
**TIMP1**	8.5	11.3	7.0	1.9×10^{-6}
**TOP2A**	5.1	6.5	2.6	3.4×10^{-4}

* P value from student's T-test

Supplementary Table S7. Expression of 16 genes in AA (n=5) and GBM (n=71) samples of the GSE4422 dataset.

Gene Name	Average of AA	Average of GBM	Fold change	P value
**CDKN3**	9.9	11.0	2.1	1.6×10^{-4}
**CHI3L1**	9.3	13.2	14.9	1.3×10^{-3}
**COL4A2**	10.2	12.4	4.6	1.4×10^{-14}
**DCN**	10.7	11.9	2.3	9.1×10^{-3}
**DLL3**	11.7	10.7	0.5	6.7×10^{-3}
**FABP7**	11.0	12.5	2.8	0.05
**IGFBP2**	9.8	13.0	9.2	1.4×10^{-4}
**IGFBP3**	10.6	12.2	3.0	3.4×10^{-3}
**LAMB1**	7.7	10.0	4.9	1.5×10^{-3}
**LGALS1**	13.1	14.1	2.0	0.02
**LGALS3**	11.9	13.4	2.8	1.2×10^{-3}
**NAMPT**	11.7	12.8	2.1	6.0×10^{-5}
**PLAT**	9.6	11.3	3.2	3.0×10^{-4}
**PTTG1**	11.5	12.8	2.5	4.6×10^{-3}
**TIMP1**	10.6	13.7	8.6	7.0×10^{-10}
**TOP2A**	8.3	10.3	4.0	0.02

* P value from student's T-test

Supplementary Figure S1.

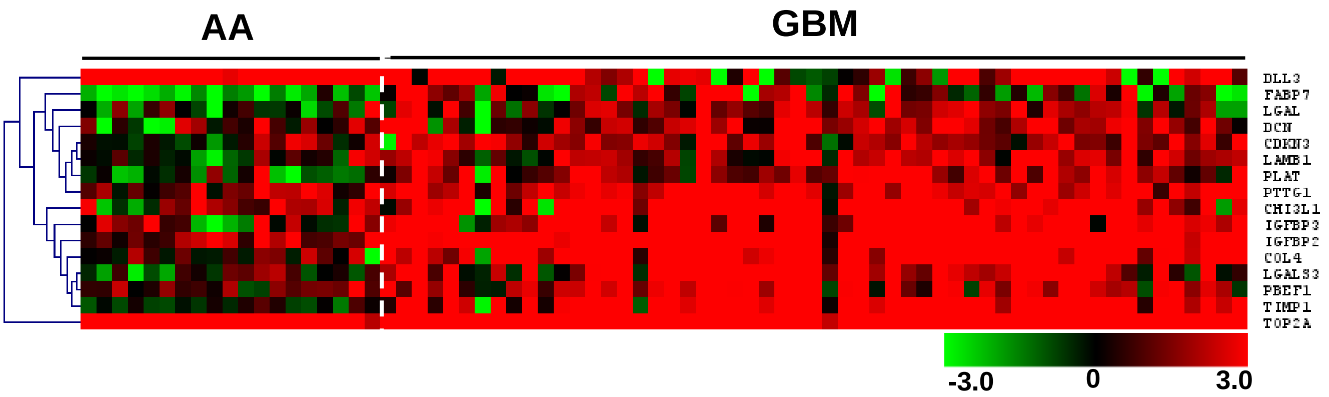

Supplementary Figure S1. Heat map of one-way hierarchical clustering of 16 PAM-identified genes in AA (n=20) and GBM (n=54) patient samples in the test set. A dual-color code was used, with red and green indicating up- and down regulation, respectively.

Supplementary Figure S2.

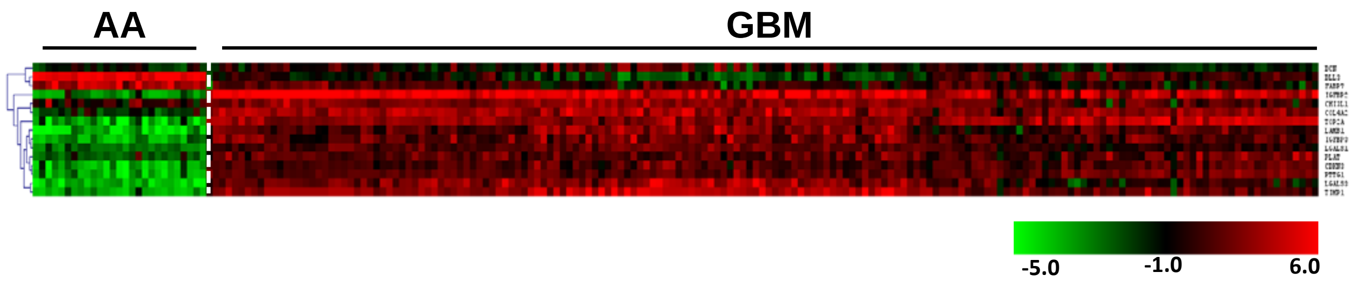

Supplementary Figure S2. Heat map of one-way hierarchical clustering of 16 PAM-identified genes in grade III glioma (n=27) and GBM (n=152) patient samples in TCGA dataset. A dual-color code was used, with red and green indicating up- and down regulation, respectively.

Supplementary Figure S3.

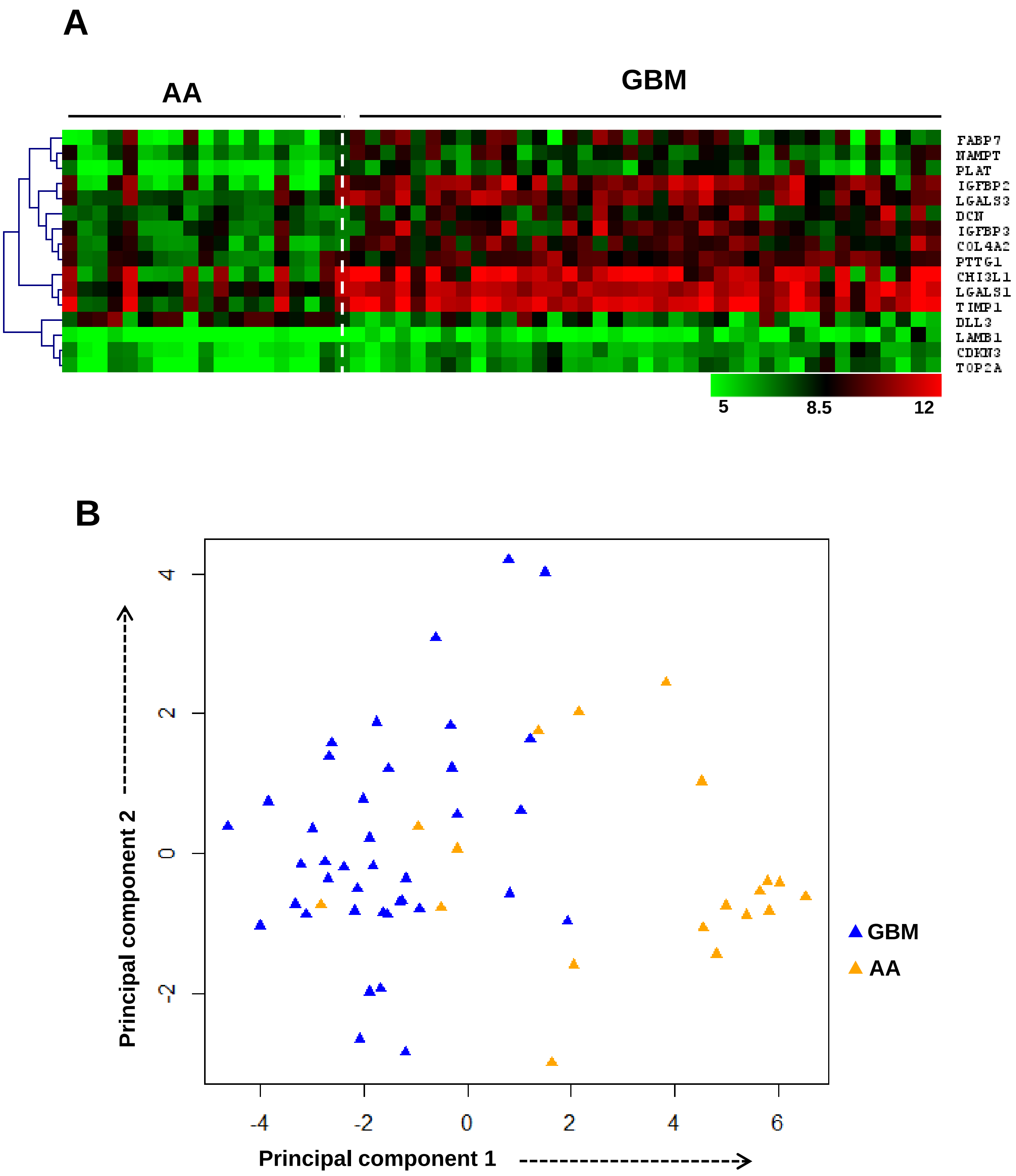

Continued…

Supplementary Figure S3.

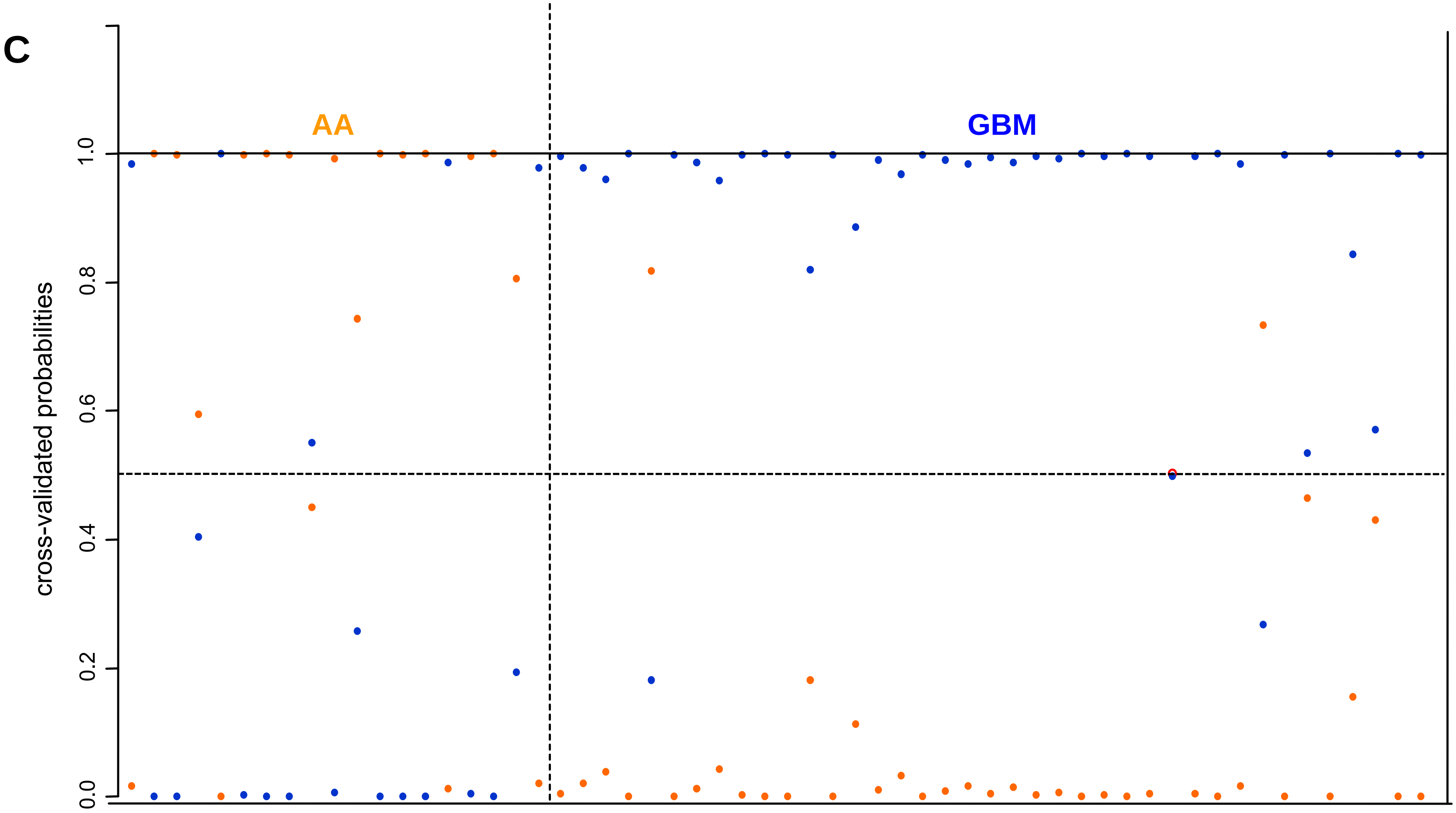

Supplementary Figure S3. A. Heat map of one-way hierarchical clustering of 16 PAM-identified genes in AA (n=19) and GBM (n=39) patient samples in GSE1993 dataset. A dual-color code was used, with red and green indicating up- and down regulation, respectively. **B.** PCA was performed using expression values of 16- PAM identified genes between AA and GBM samples in GSE1993 dataset. A scatter plot is generated using the first two principal components for each sample. The color of the samples is as indicated. **C.** The detailed probabilities of 10-fold cross-validation for the samples of GSE1993 dataset based on the expression values of 16 genes are shown. For each sample, its probability as AA (orange color) and GBM (blue color) are shown and it was predicted by the PAM program as either AA or GBM based on which grade's probability is higher. The original histological grade of the samples is shown on the top.

Supplementary Figure S4.

A

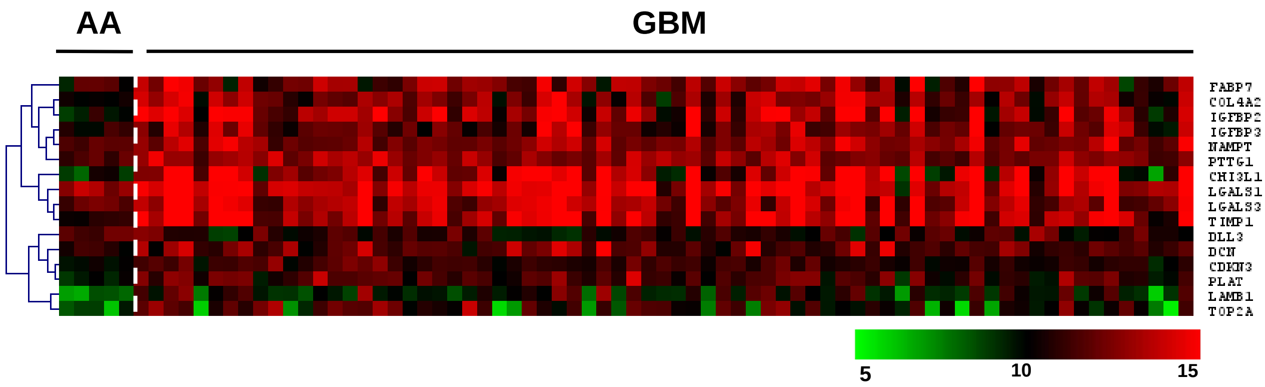

B

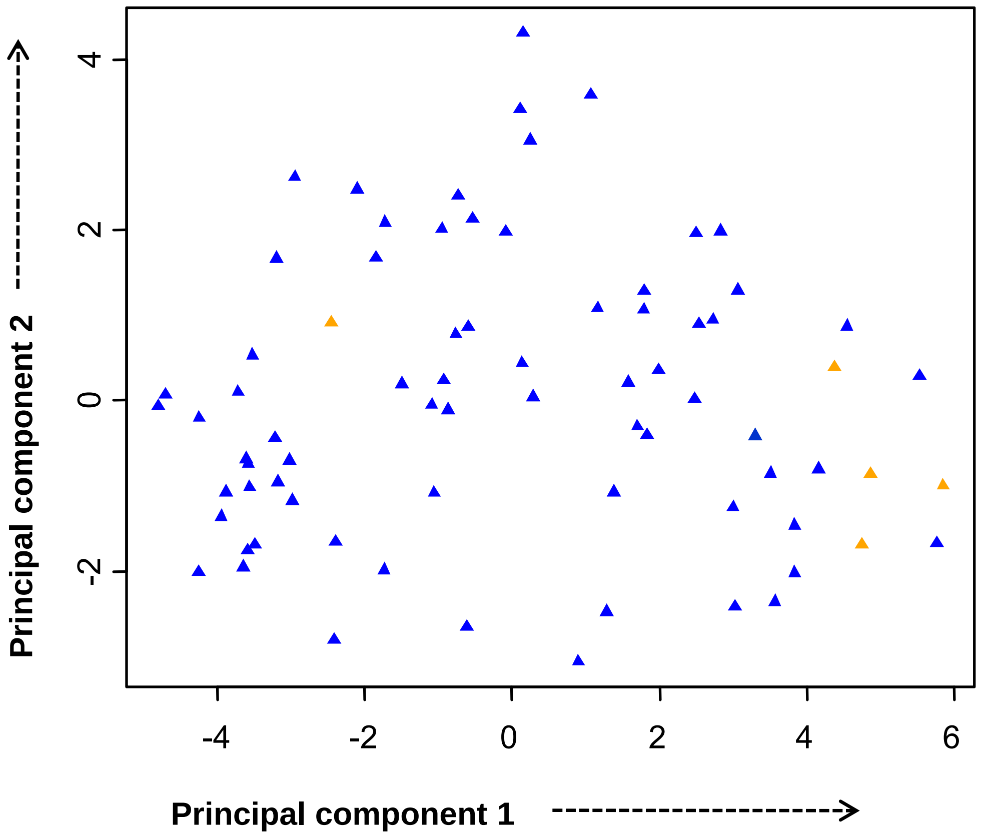

Continued…

Supplementary Figure S4.

C

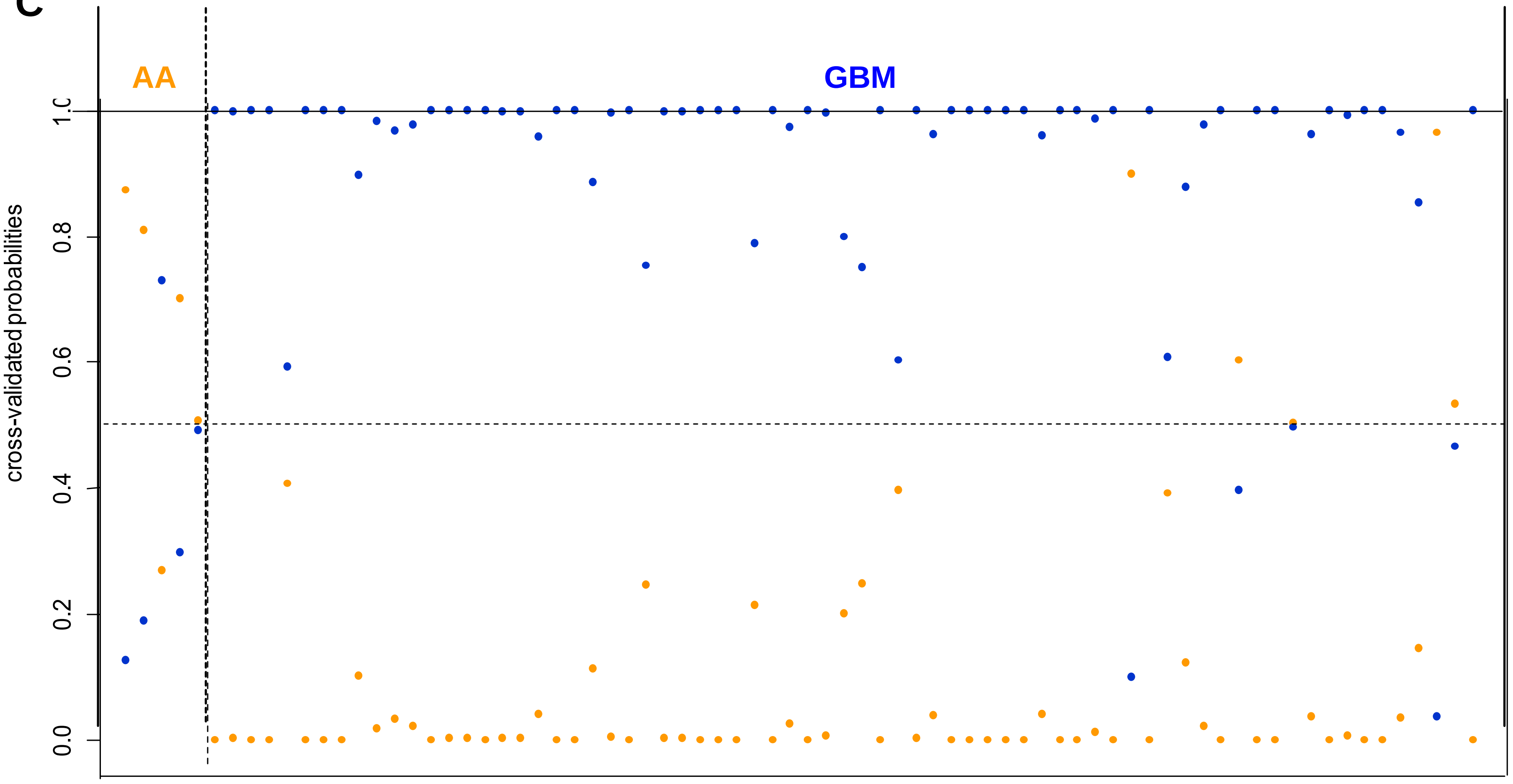

Supplementary Figure S4. A. Heat map of one-way hierarchical clustering of 16 PAM-identified genes in AA (n=5) and GBM (n=71) patient samples in GSE4422 dataset. A dual-color code was used, with red and green indicating up- and down regulation, respectively. **B.** PCA was performed using expression values of 16- PAM identified genes between AA and GBM samples in GSE1993 dataset. A scatter plot is generated using the first two principal components for each sample. The color of the samples is as indicated. **C.** The detailed probabilities of 10-fold cross-validation for the samples of GSE4422 dataset based on the expression values of 16 genes are shown. For each sample, its probability as AA (orange color) and GBM (blue color) are shown and it was predicted by the PAM program as either AA or GBM based on which grade's probability is higher. The original histological grade of the samples is shown on the top.

Supplementary Figure S5.

A

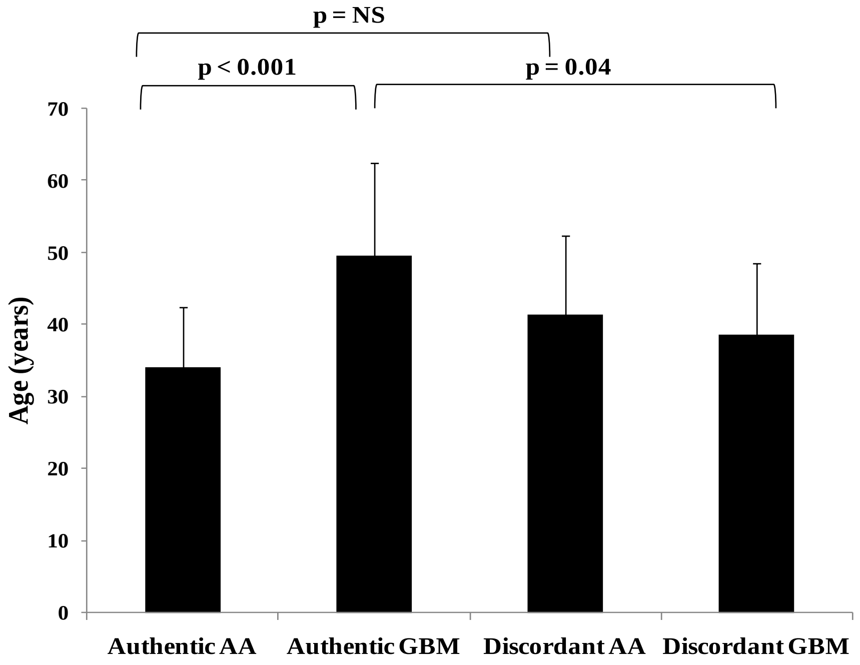

B

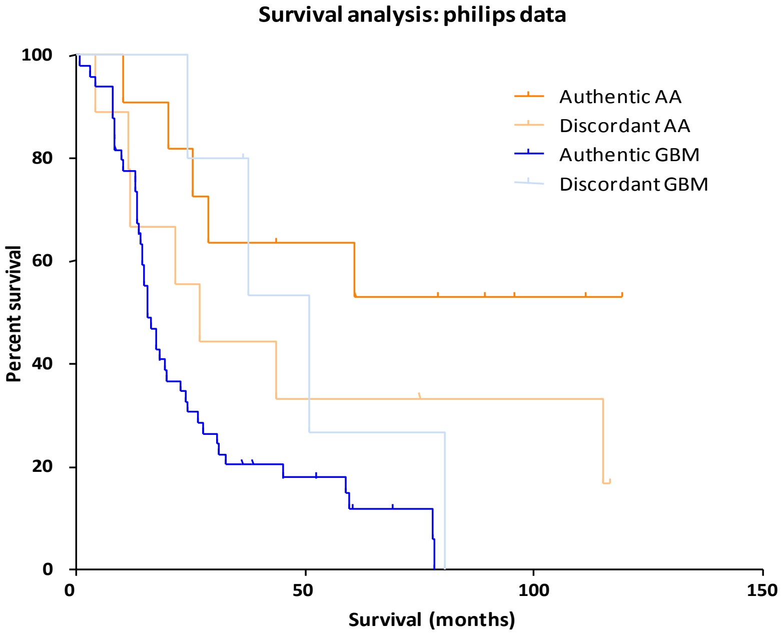

Supplementary Figure S5. A. The detailed probabilities of 10-fold cross-validation for the samples of GSE4271 dataset based on the expression values of 16 genes are shown. For each sample, its probability as AA (orange color) and GBM (blue color) are shown and it was predicted by the PAM program as either AA or GBM based on which grade's probability is higher. The original histological grade of the samples is shown on the top. **B.** The average Age at Diagnosis along with standard deviation is plotted for Authentic AAs, (n=12), Authentic GBMs (n=68), Discordant AAs (n=10) and Discordant GBMs (n=8) of GSE4271 dataset. **C**. The Kaplan Meier survival analysis of samples of GSE4271 dataset.

Supplementary Figure S6.

A

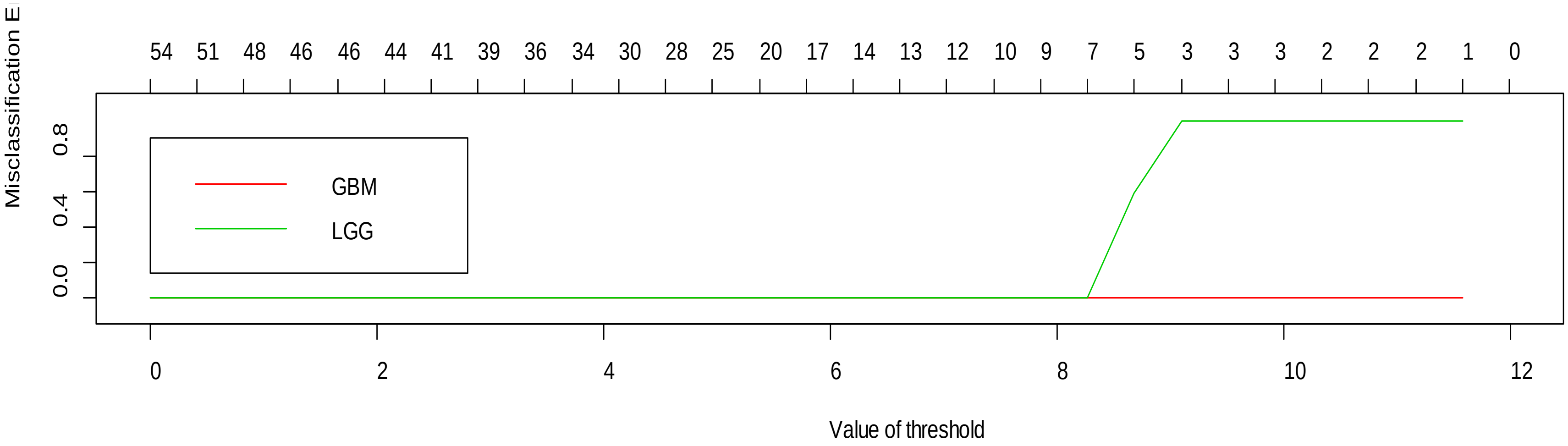

Supplementary Figure S6.

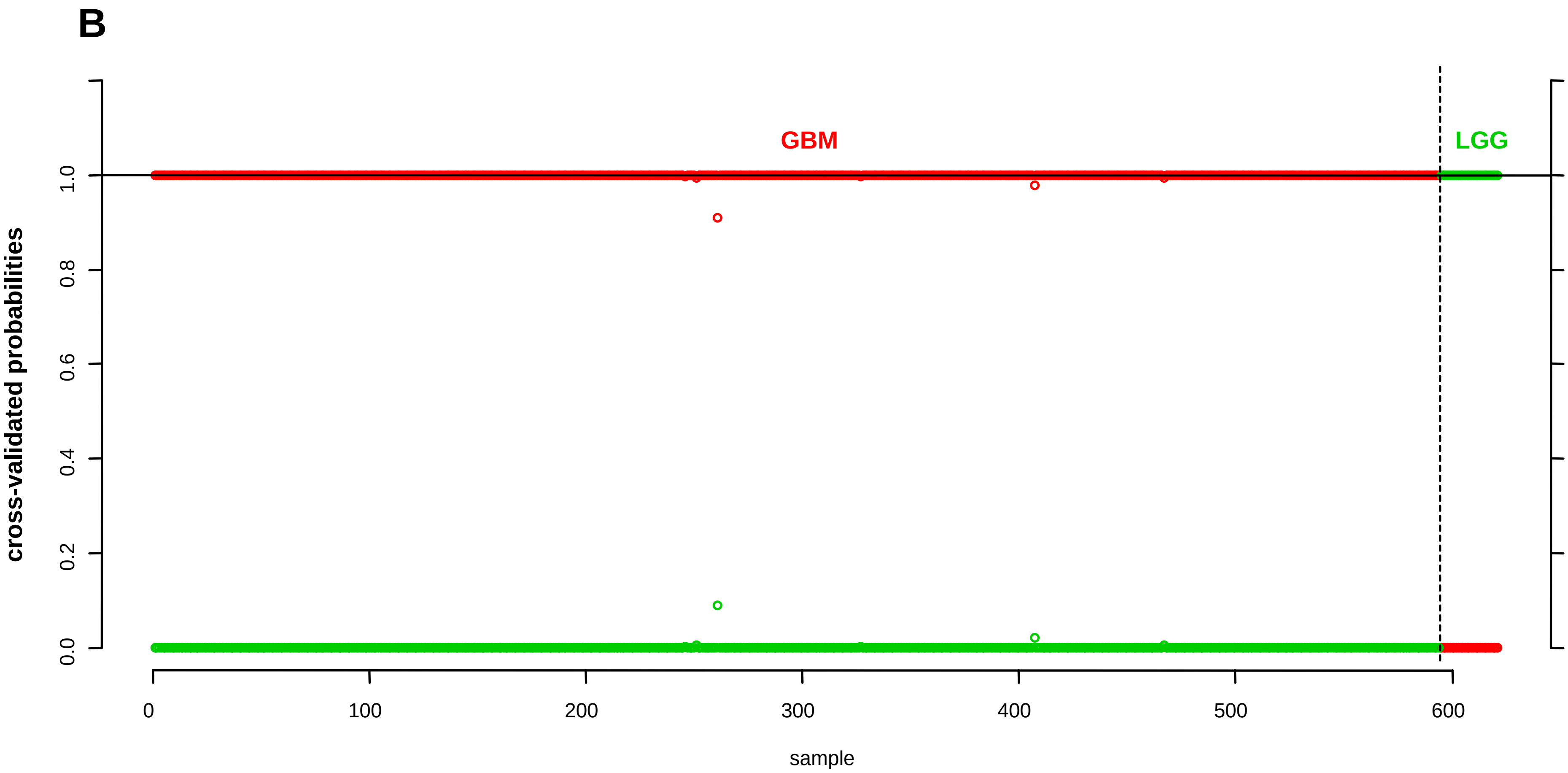

Supplementary Figure S6. PAM analysis of the Petalidis-gene signature in TCGA dataset. A. Plot showing classification error for the Petalidis geneset in TCGA dataset. The threshold value of 0.0 corresponded to all 54 genes which classified AA (n=27) and GBM (n=604) samples with classification error of 0.000. **B.** The detailed probabilities of 10-fold cross-validation for the samples of TCGA dataset based on Petalidis geneset are shown. For each sample, its probability as AA (green color) and GBM (red color) are shown and it was predicted by the PAM program as either AA or GBM based on which grade's probability is higher. The original histological grade of the samples is shown on the top.

Supplementary Figure S7.

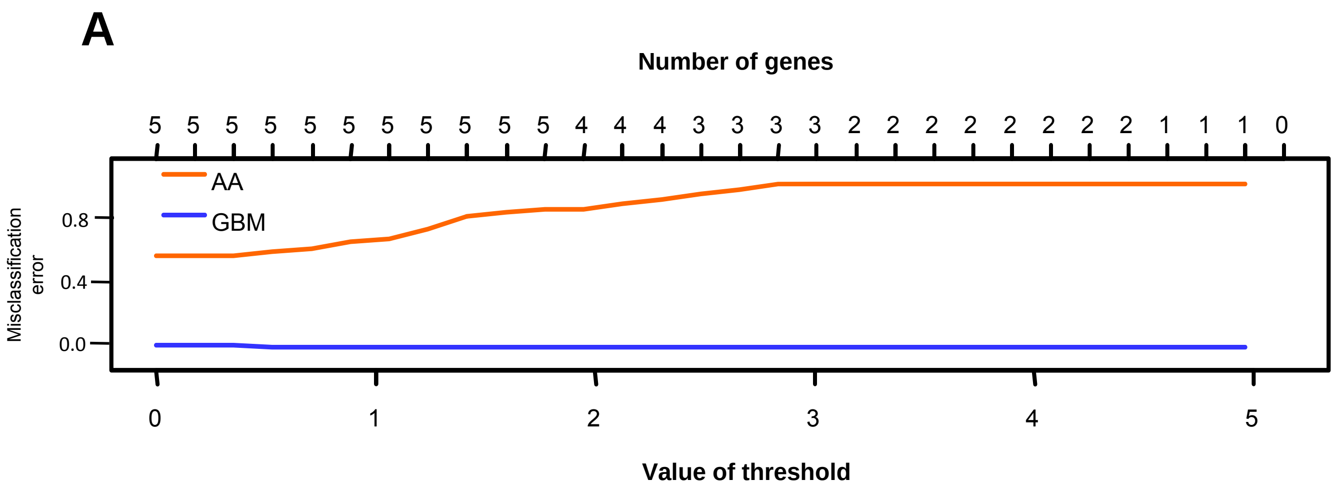

Supplementary Figure S7.

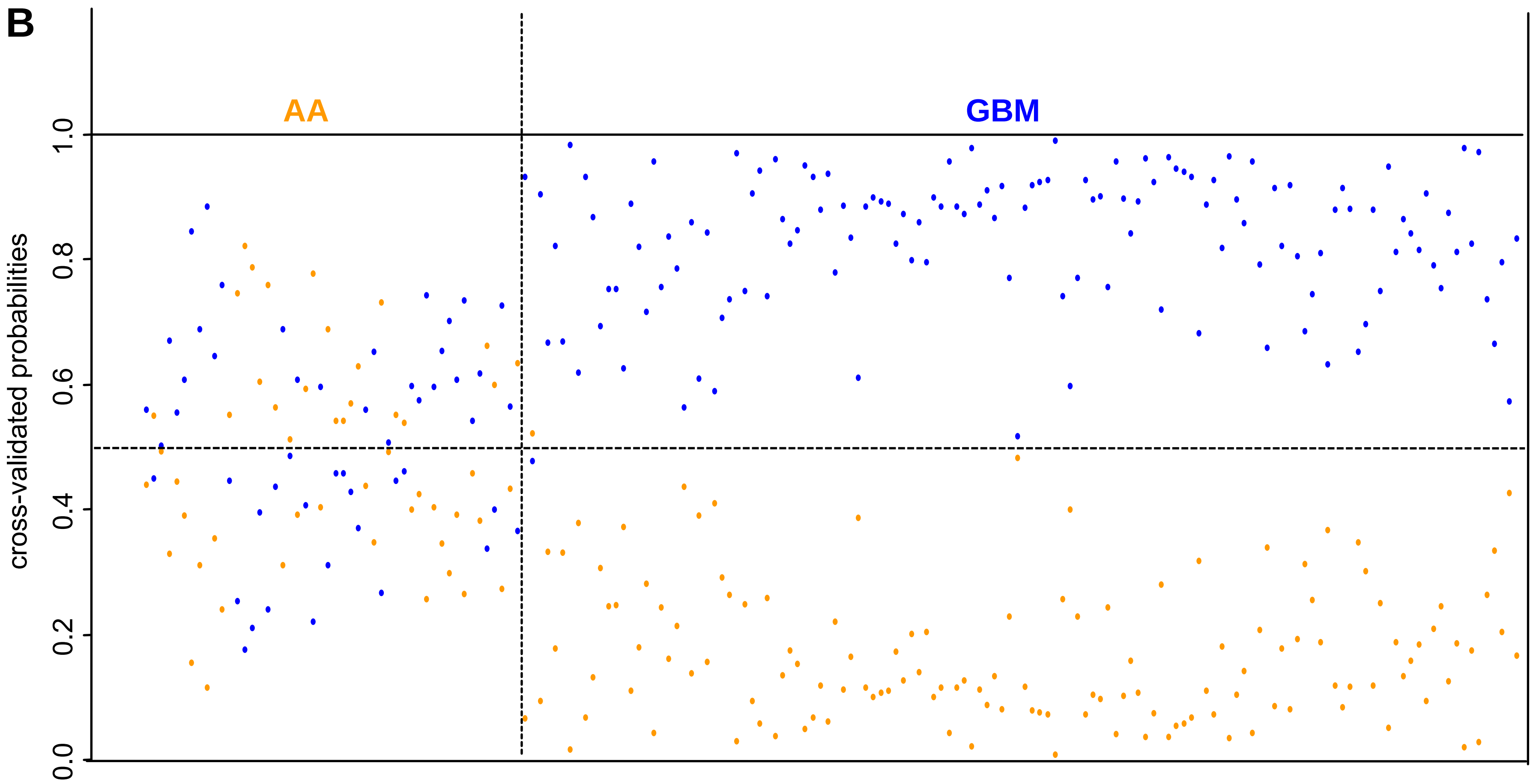

Supplementary Figure S7. PAM analysis of the Phillips gene signature in our dataset. A. Plot showing classification error for the Phillips gene set in our dataset. The threshold value of 0.0 that correspond to all 5 genes which classified AA (n=50) and GBM (n=132) samples with classification error of 0.159. **B.** The detailed probabilities of 10-fold cross-validation for the samples of our dataset based on Phillips gene set are shown. For each sample, its probability as AA (orange color) and GBM (blue color) are shown and it was predicted by the PAM program as either AA or GBM based on which grade's probability is higher. The original histological grade of the samples is shown on the top.

Supplementary Figure S8.

A

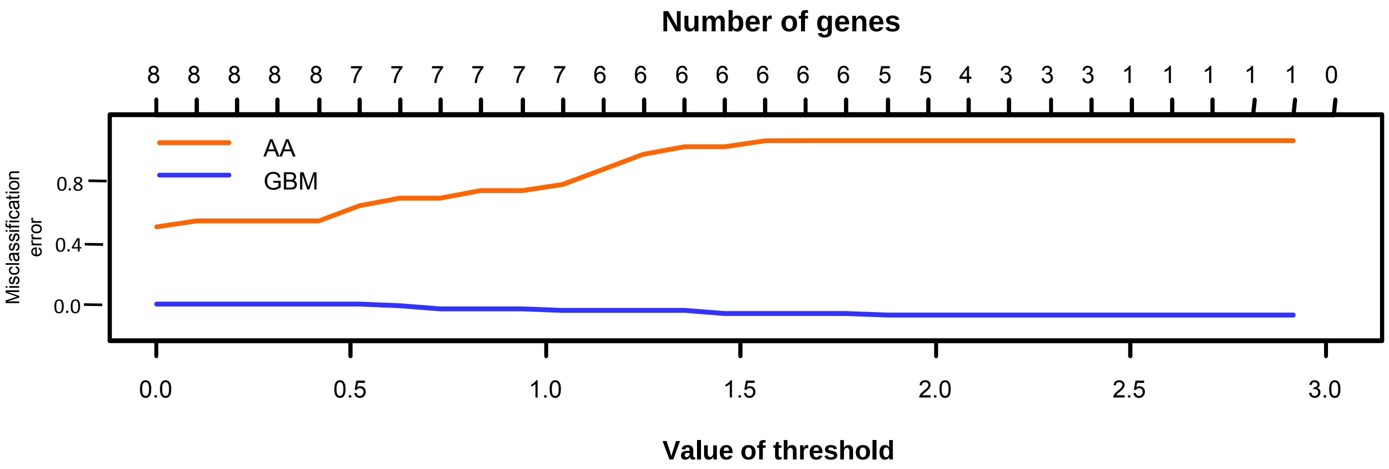

Supplementary Figure S8.

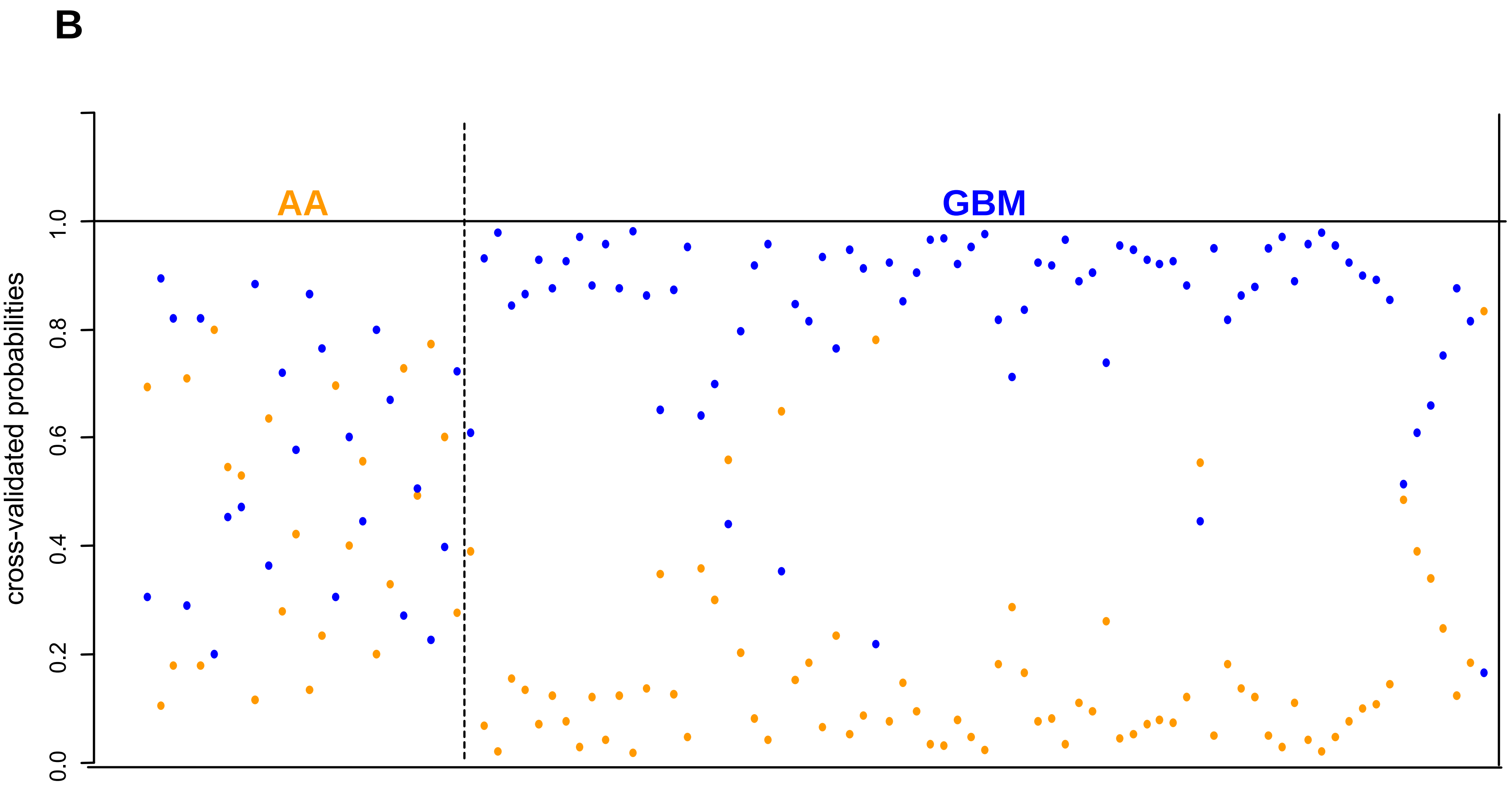

Supplementary Figure S8. PAM analysis of the Phillips-gene signature in Phillips dataset. A. Plot showing classification error for the Phillips gene set in Phillips dataset. The threshold value of 0.0 that correspond to all 8 genes, which classified AA (n=24) and GBM (n=76) samples with classification error of 0.169. **B.** The detailed probabilities of 10-fold cross-validation for the samples of Phillips dataset are shown. For each sample, its probability as AA (orange color) and GBM (blue color) are shown and it was predicted by the PAM program as either AA or GBM based on which grade's probability is higher. The original histological grade of the samples is shown on the top.

Supplementary Figure S9

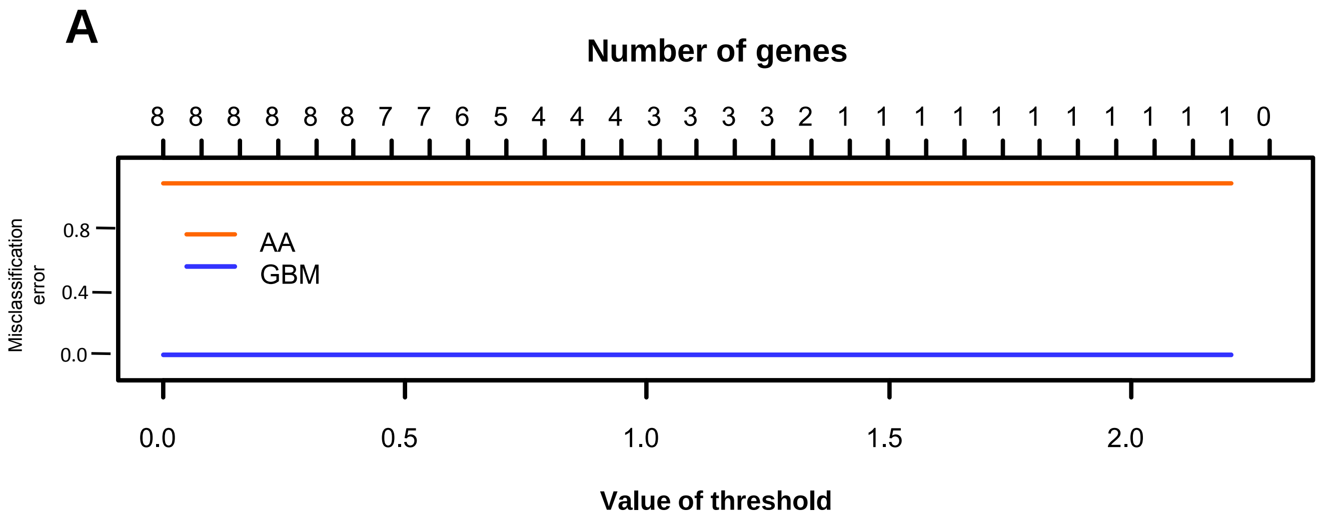

Supplementary Figure S9

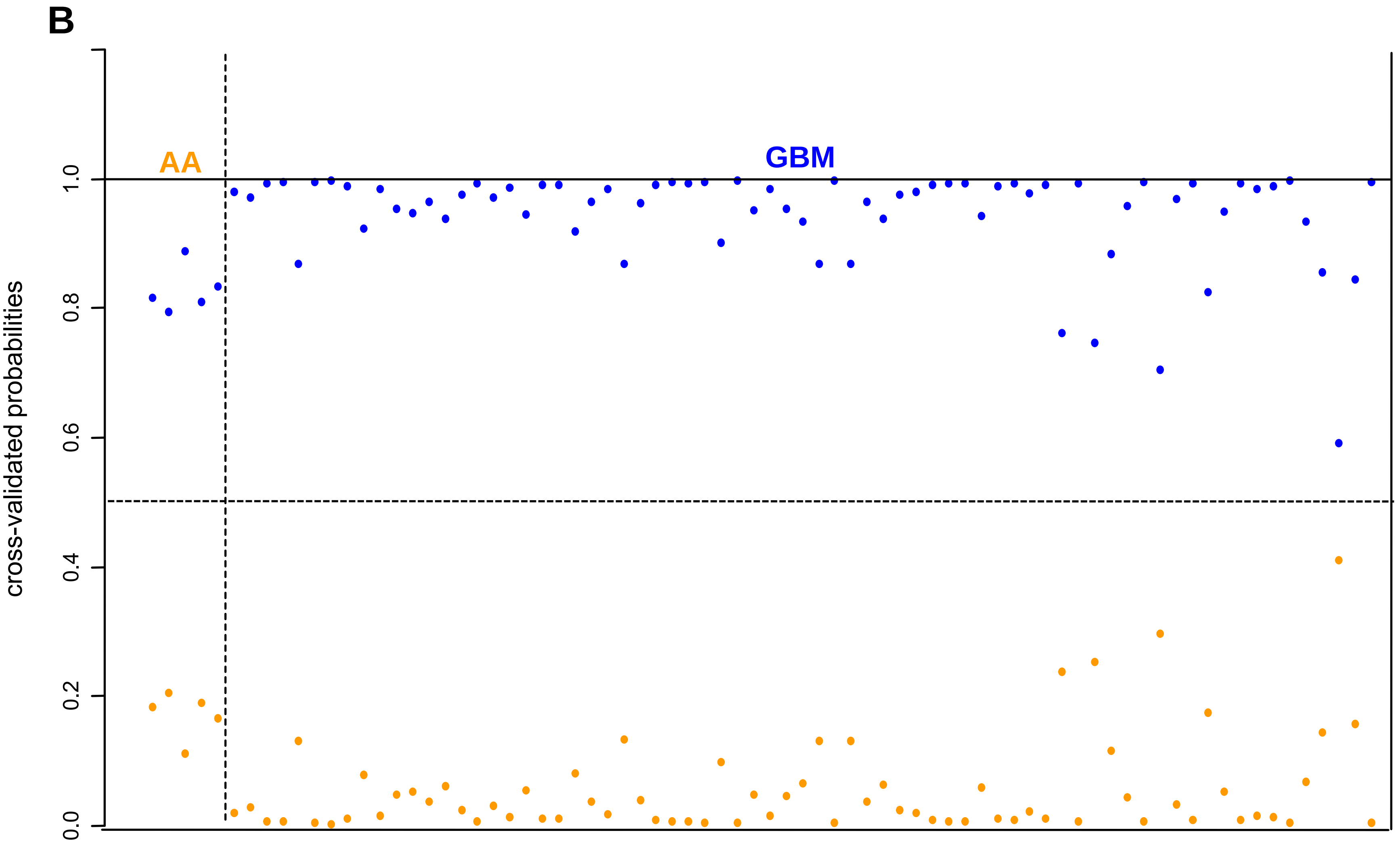

Supplementary Figure S9. PAM analysis of the Phillips-gene signature in GSE4422 dataset A. Plot showing classification error for the Phillips gene set in GSE4422 dataset. The threshold value of 0.0 corresponded to all 8 genes which classified AA (n=5) and GBM (n=76) samples with classification error of 0.065. **B.** The detailed probabilities of 10-fold cross-validation for the samples of GSE4422 dataset based on Phillips gene signature are shown. For each sample, its probability as AA (orange color) and GBM (blue color) are shown and it was predicted by the PAM program as either AA or GBM based on which grade's probability is higher. The original histological grade of the samples is shown on the top.

Supplementary Figure S10.

A

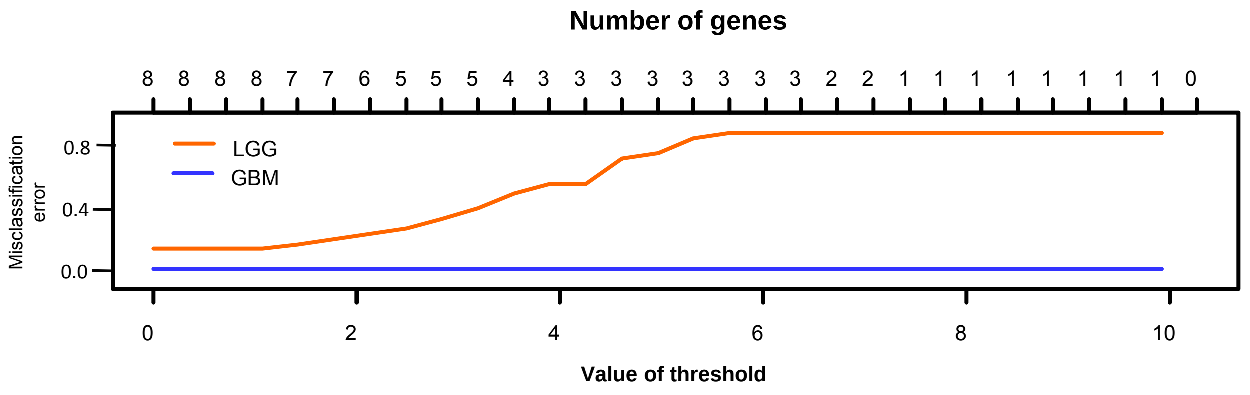

Supplementary Figure S10.

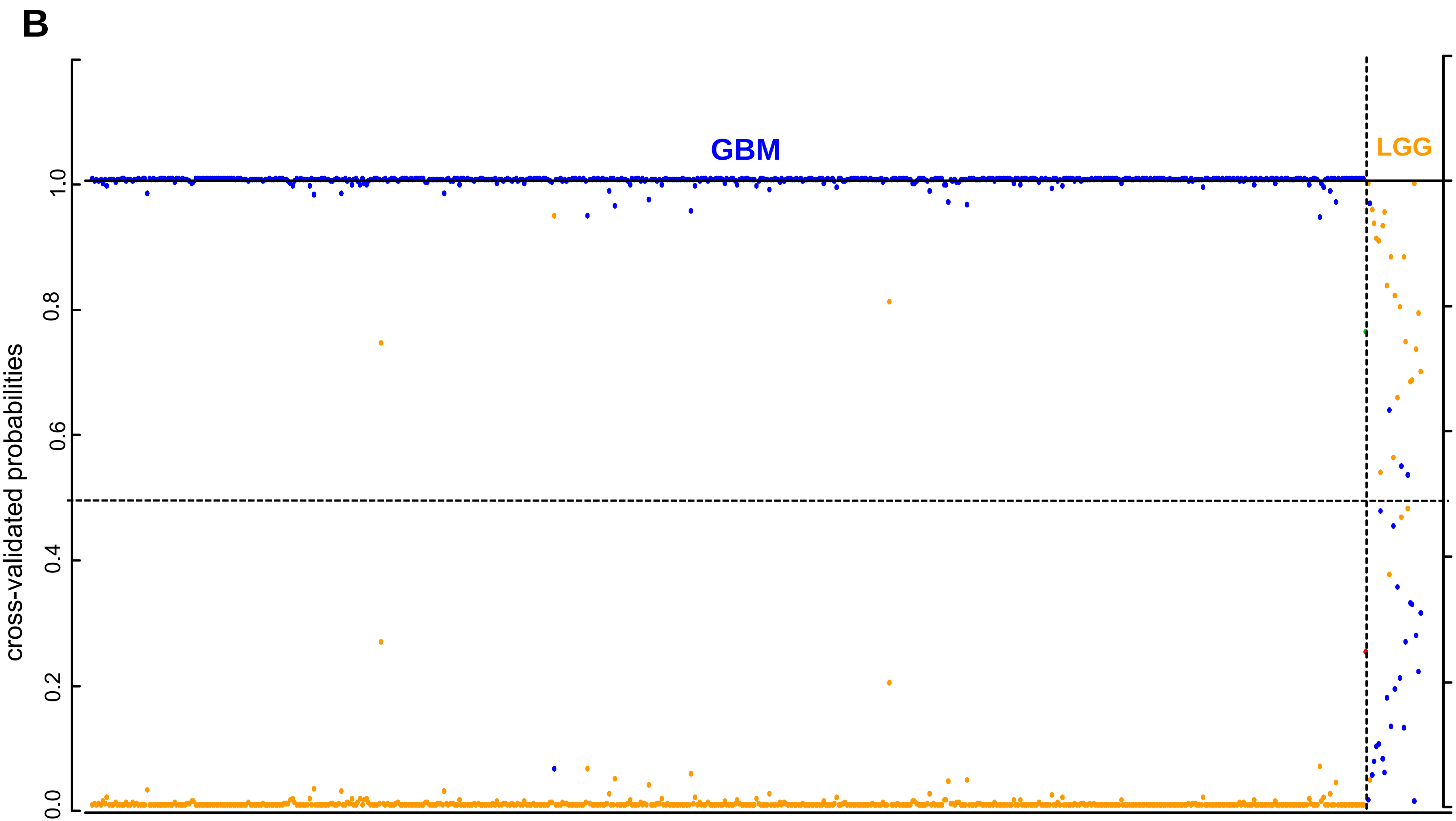

Supplementary Figure S10. PAM analysis of the Phillips-gene signature in TCGA dataset. A. Plot showing classification error for the Phillips gene set in TCGA dataset. The threshold value of 0.0 corresponded to all 8 genes which classified AA (n=27) and GBM (n=604) samples with classification error of 0.008. **B.** The detailed probabilities of 10-fold cross-validation for the samples of TCGA dataset based on Phillips gene set are shown. For each sample, its probability as AA (orange color) and GBM (blue color) are shown and it was predicted by the PAM program as either AA or GBM based on which grade's probability is higher. The original histological grade of the samples is shown on the top.

Supplementary Figure S11

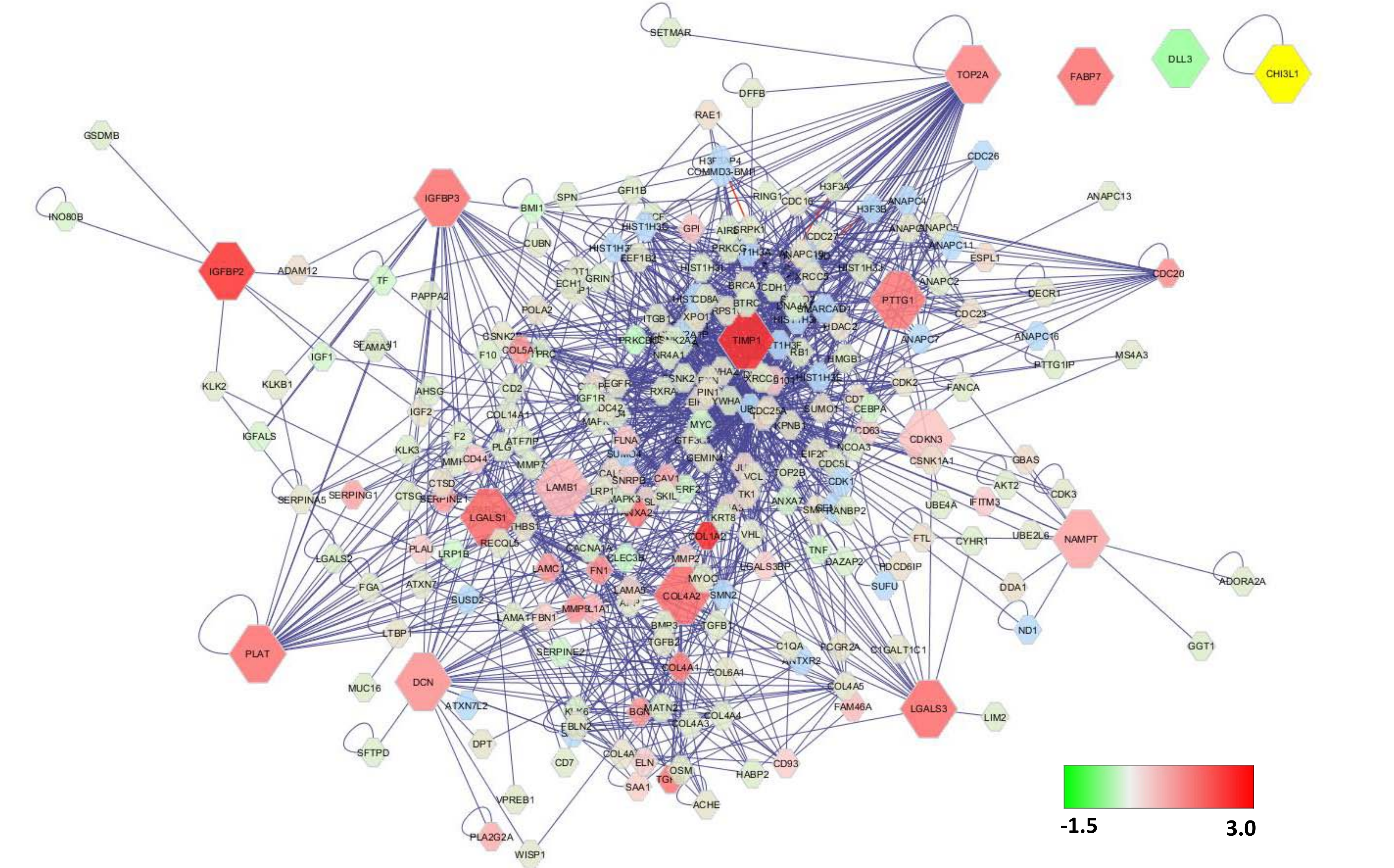

Supplementary Figure S11. Network obtained by using 16-genes of classification signature as input genes to Bisogenet plugin in Cytoscape. The generated network had 252 nodes (genes) and 1498 edges (interactions between genes/proteins). This network consisted of the seed proteins with their immediate interacting neighbors. The nodes corresponding to the input genes are highlighted by the bigger node size as compared to the rest of the interacting partners. The color code is as indicated in the scale.
